# Serotonin Signaling Through the 5-HT_1B_ Receptor and NADPH Oxidase 1 in Pulmonary Arterial Hypertension

**DOI:** 10.1161/ATVBAHA.116.308929

**Published:** 2017-06-21

**Authors:** Katie Y. Hood, Kirsty M. Mair, Adam P. Harvey, Augusto C. Montezano, Rhian M. Touyz, Margaret R. MacLean

**Affiliations:** From the Institute of Cardiovascular and Medical Sciences, BHF Glasgow Cardiovascular Research Centre, University of Glasgow, United Kingdom.

**Keywords:** hypertension, pulmonary, models, animal, NADPH oxidase, receptor, serotonin, 5-HT_1B_, serotonin

## Abstract

**Objective—:**

Serotonin can induce human pulmonary artery smooth muscle cell (hPASMC) proliferation through reactive oxygen species (ROS), influencing the development of pulmonary arterial hypertension (PAH). We hypothesize that in PASMCs, serotonin induces oxidative stress through NADPH-oxidase–derived ROS generation and reduced Nrf-2 (nuclear factor [erythroid-derived 2]-like 2) antioxidant systems, promoting vascular injury.

**Approach and Results—:**

HPASMCs from controls and PAH patients, and PASMCs from Nox1^−/−^ mice, were stimulated with serotonin in the absence/presence of inhibitors of Src kinase, the 5-HT_1B_ receptor, and NADPH oxidase 1 (Nox1). Markers of fibrosis were also determined. The pathophysiological significance of our findings was examined in vivo in serotonin transporter overexpressing female mice, a model of pulmonary hypertension. We confirmed thatserotonin increased superoxide and hydrogen peroxide production in these cells. For the first time, we show that serotonin increased oxidized protein tyrosine phosphatases and hyperoxidized peroxiredoxin and decreased Nrf-2 and catalase activity in hPASMCs. ROS generation was exaggerated and dependent on cellular Src-related kinase, 5-HT_1B_ receptor, and the serotonin transporter in human pulmonary artery smooth muscle cells from PAH subjects. Proliferation and extracellular matrix remodeling were exaggerated in human pulmonary artery smooth muscle cells from PAH subjects and dependent on 5-HT_1B_ receptor signaling and Nox1, confirmed in PASMCs from Nox1^−/−^ mice. In serotonin transporter overexpressing mice, SB216641, a 5-HT_1B_ receptor antagonist, prevented development of pulmonary hypertension in a ROS-dependent manner.

**Conclusions—:**

Serotonin can induce cellular Src-related kinase–regulated Nox1-induced ROS and Nrf-2 dysregulation, contributing to increased post-translational oxidative modification of proteins and activation of redox-sensitive signaling pathways in hPASMCs, associated with mitogenic responses. 5-HT_1B_ receptors contribute to experimental pulmonary hypertension by inducing lung ROS production. Our results suggest that 5-HT_1B_ receptor–dependent cellular Src-related kinase-Nox1-pathways contribute to vascular remodeling in PAH.

## Abstract 2

Supplemental Digital Content is available in the text.

Serotonin has been implicated in the pathogenesis of pulmonary arterial hypertension (PAH)^1–3^ and has been recognized as a potent naturally occurring pulmonary vasoconstrictor^4^ and smooth muscle cell mitogen.^2^ Serotonin promotes pulmonary artery (PA) remodeling and proliferation of human PA smooth muscle cells (hPASMCs) via the 5-HT_1B_ receptor (5-HT_1B_R) and the serotonin transporter (SERT).^5–8^ Serotonin can also cause constriction of human and rodent PAs via the 5-HT_1B_R.^4,9^

Reactive oxygen species (ROS), produced primarily by the NADPH oxidase (Nox) family of enzymes in the vasculature, induce oxidative stress and play a critical role in oxidative damage to proteins, lipids, and DNA.^10^ Altered redox signaling and increased ROS bioavailability have been implicated in chronic diseases, including PAH.^11,12^ Excessive amounts of ROS in PAs can oxidize and inactivate signaling molecules, such as protein tyrosine phosphatases (PTPs), or can drive irreversible protein modification through addition of carbonyl groups on protein side chains, a marker for oxidative stress.^12,13^

Intracellular ROS levels are regulated by the balance between ROS-generating enzymes, such as Noxs, and antioxidant enzymes that include superoxide dismutases, catalase, and the peroxiredoxin systems,^14^ which are regulated by a key transcription factor Nrf-2 (nuclear factor [erythroid-derived 2]-like 2). Nrf-2 activators attenuate experimental pulmonary hypertension (PH).^15^ Increased expression of Nox isoforms 1 and 4 in PAs has been demonstrated in experimental models of PH and in PASMCs from PAH patients.^12^ Cellular Src-related kinase (c-Src) is the predominant nonreceptor tyrosine kinase in the vasculature, which is required for regulation of Nox activity,^16^ and this may be dysregulated in PAs of PAH patients and experimental PH.^17^

Serotonin-induced ROS has been implicated in the proliferative response of proximal bovine and murine PASMCs.^18,19^ However, it is unclear whether serotonin influences ROS in hPASMC and is the focus of our study.

Although studies have shown that serotonin promotes PA remodeling mainly through SERT and 5-HT_1B_R,^4,7,20^ the role of Nox isoforms in serotonin-dependent ROS production, antioxidant regulation, and redox-sensitive processes downstream of ROS production has yet to be investigated. It is important to investigate this in the distal hPASMCs that contribute to the pathophysiology of PAH. For the first time, we investigate the role of serotonin in Nox-derived ROS in hPASMCs, specifically, Nox1-derived ROS in serotonin-induced Nrf-2 dysfunction, protein carbonylation, and oxidation of antioxidant and signaling molecules, peroxiredoxin, and PTPs.

## Materials and Methods

Materials and Methods are available in the online-only Data Supplement.

## Results

### Serotonin Increases ROS Production

**Figure 1.**
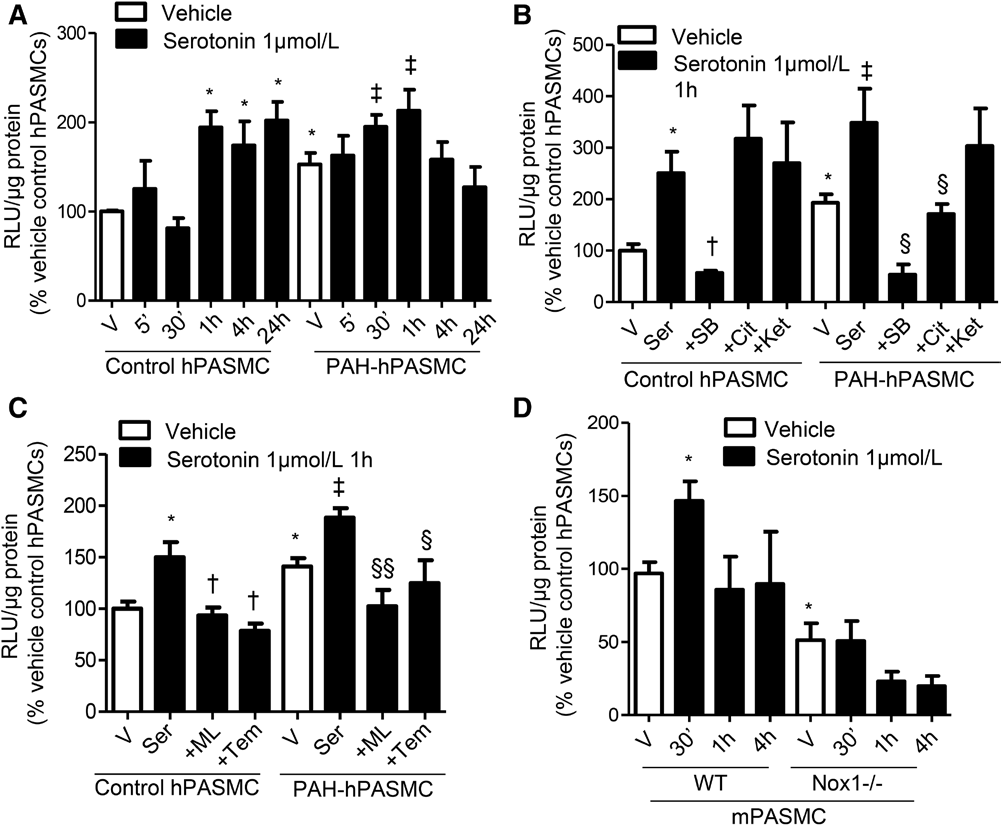
Serotonin increases reactive oxygen species (ROS) production through Nox-dependent mechanisms. Time-dependent increase of ROS production by serotonin (1 µmol/L) in control human pulmonary artery smooth muscle cells (hPASMCs) and pulmonary arterial hypertension (PAH)-hPASMCs assessed by lucigenin-enhanced chemiluminescence (**A**). hPASMCs and human pulmonary artery smooth muscle cells from PAH subjects (PAH-hPASMCs) were exposed to serotonin for the time of peak ROS production (1 h) previously shown in (**A**), in the presence or absence of inhibitors of Nox1 (ML171, 1 µmol/L) and superoxide dismutase mimetic Tempol (10 µmol/L; **B**). Serotonin-induced ROS production in wild-type (WT) and Nox1^−/−^ mouse pulmonary artery smooth muscle cells (mPASMCs; **C**). ROS production by serotonin (1 µmol/L) in control hPASMCs and PAH-hPASMCs at 1 h stimulation, in the presence or absence of 5-HT_1B_R antagonist, SB224289, SERT inhibitor, citalopram (Cit), or 5-HT_2A/1D_R antagonist (**D**). Data are expressed as relative light units (RLU)/µg protein, expressed as percentage of vehicle control conditions. Results are mean±SEM of 5 experiments, in triplicate. *P<0.05 vs vehicle control hPASMC or vehicle WT mPASMCs; †*P*<0.05 vs treated control hPASMC; ‡*P*<0.05 vs vehicle PAH-hPASMCs; §*P*<0.05, §§*P*<0.01 vs treated PAH-hPASMCs determined by ANOVA with Tukey post hoc test. Ket indicates ketanserin; ML, ML171; SB, SB224289; Ser, serotonin; Tem, Tempol; and V, vehicle.

Basal ROS production was higher in human pulmonary artery smooth muscle cells from PAH subjects (PAH-hPASMCs) compared with hPASMCs. In hPASMCs, serotonin increased ^.^O_2_^−^ production at 1, 4, and 24 hours of stimulation, whereas in PAH-hPASMCs serotonin increased ^.^O_2_^−^ generation more rapidly at 30 minutes and 1 hour (Figure 1A). In control hPASMCs, serotonin-induced ^.^O_2_^−^ generation was blocked by 5-HT_1B_R antagonist, SB224289, but not a SERT antagonist (citalopram) or a 5-HT_1D/2A_R inhibitor (ketanserin). In PAH-hPASMCs, both the 5-HT_1B_R and the SERT mediate ^.^O_2_^−^ generation as SB224289 and citalopram blocked the effects of serotonin (Figure 1B). No effects were observed with serotonin receptor antagonists alone (data not shown).

**Figure 2.**
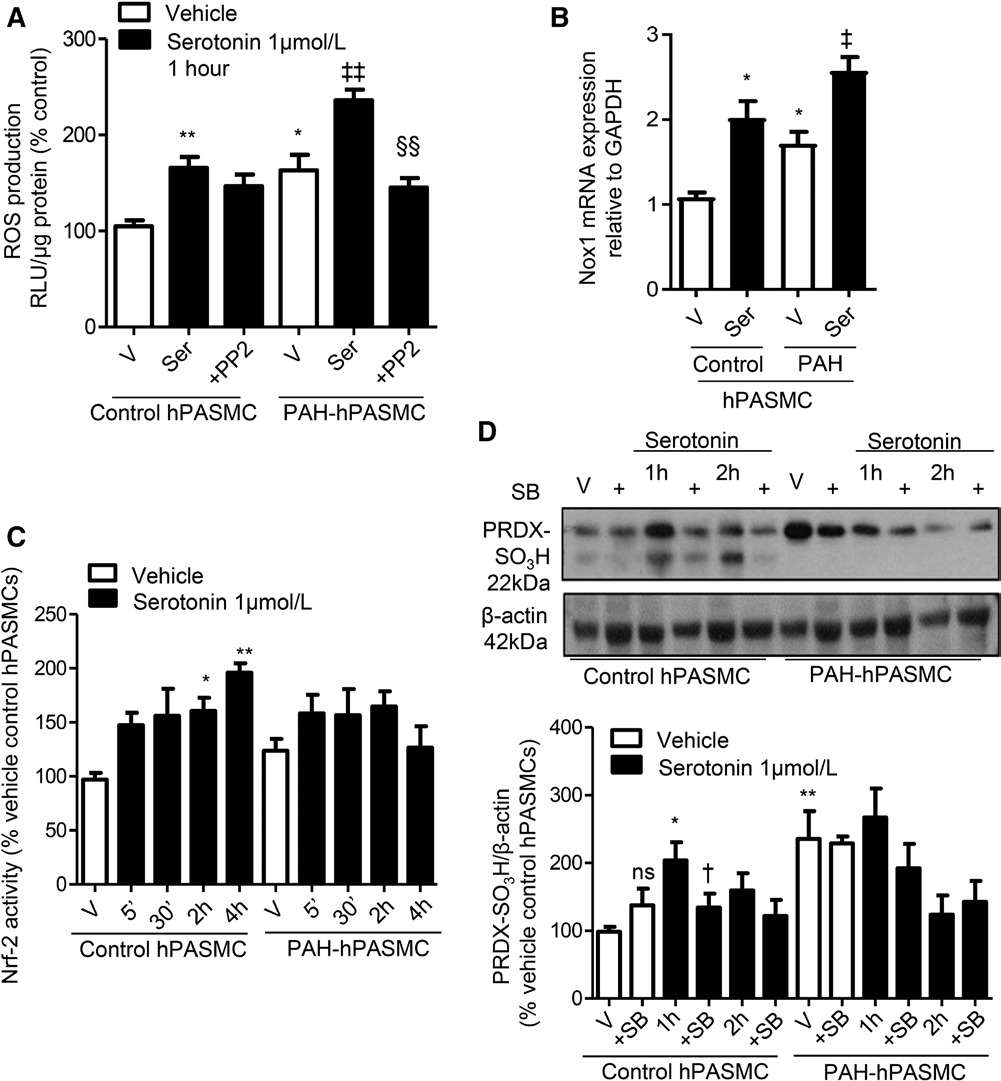
Effect of serotonin on Nrf-2 activation and oxidation of peroxiredoxin. Serotonin-induced reactive oxygen species (ROS) production in the presence or absence of Src kinase inhibitor, PP2 (10 µmol/L) in control human pulmonary artery smooth muscle cell (hPASMC) and pulmonary arterial hypertension (PAH)-hPASMCs. Data are expressed as relative light units (RLU)/µg protein, expressed as percentage of vehicle control conditions (**A**). Transcript levels of Nox1 (**B**) in response to serotonin. Nuclear translocation of Nrf-2 by serotonin at 5 min to 4 h was assessed as an indicator of Nrf-2 activity. Data are expressed as RLU/µg protein expressed as percentage of vehicle control conditions (**C**). Effects of serotonin (1–2 h) in the presence or absence of the 5-HT_1B_R antagonist, SB224289, on protein expression of hyperoxidized peroxiredoxin, hyperoxidized peroxiredoxin (PRDX-SO_3_H) in hPASMCs (**D**). Results are mean±SEM of 3 to 6 experiments. Graph represents mRNA expression relative to GAPDH. *P<0.05, **P<0.01 vs vehicle control hPASMC or wild-type (WT) vehicle mouse pulmonary artery smooth muscle cells (mPASMCs); †*P*<0.05 vs serotonin 1 h control hPASMCs; vehicle human pulmonary artery smooth muscle cells from PAH subjects (PAH-hPASMCs; Nox1); ‡*P*<0.05, ‡‡ *P*<0.01 vs vehicle PAH-hPASMCs; §§*P*<0.01 vs serotonin-treated PAH-hPASMCs determined by ANOVA with Tukey post hoc test. ns indicates not significant; SB, SB224289; Ser, serotonin; and V, vehicle.

The Nox1 inhibitor ML171, and the radical scavenger tempol, inhibited serotonin-induced ·O_2_^−^ formation in control and PAH-hPASMCs (Figure 1C). To control for nonspecific effects of ML171, we examined ROS production in mouse PASMCs (mPASMCs) derived from wild-type (WT) and Nox1^−/−^ mice. Serotonin-induced ROS production was observed in WT mPASMCs but absent in Nox1^−/−^ mPASMCs at 30 minutes’ stimulation (Figure 1D).

c-Src may be involved in transactivation and phosphorylation of subunits required for Nox assembly^21^ and is a proximal regulator of vascular Nox activation.^16^ Exposure of PAH-hPASMCs to a Src kinase inhibitor, PP2, inhibited serotonin-induced ROS in PAH-hPASMCs while having no effect in control hPASMCs (Figure 2A).

Hydrogen peroxide (H_2_O_2_) is a common substrate for the peroxidation reaction of peroxiredoxins.^22^ Basal intracellular H_2_O_2_ levels, assessed by Amplex red, were increased in PAH-hPASMCs versus controls. Serotonin caused H_2_O_2_ production at 5 and 30 minutes in control hPASMCs, whereas H_2_O_2_ production in PAH-hPASMCs was more sustained from 30 minutes to 4 hours (Figure IA in the online-only Data Supplement). In control hPASMCs, serotonin reduced catalase activity to similar levels observed in the PAH-hPASMCs, whereas in PAH-hPASMCs, serotonin further reduced activity of catalase after 5 minutes (Figure IB in the online-only Data Supplement). In line with accumulation of H_2_O_2_, there was a reduction in basal catalase activity in PAH versus control hPASMCs (Figure IB in the online-only Data Supplement). In addition, catalase activity was reduced by serotonin treatment (30 minutes to 2 hours) in control hPASMCs. In PAH-hPASMCs, the already lowered basal catalase activity was further attenuated by serotonin more rapidly than in the control cells, at 5 minutes’ stimulation (Figure IB in the online-only Data Supplement). In support, glutathione mRNA levels were significantly attenuated in PAH-hPASMCs versus control hPASMCs (Figure IC in the online-only Data Supplement).

### Regulation of Nox Isoforms by Serotonin in hPASMCs

Basal gene expression of Nox1 was increased in PAH-hPASMCs compared with controls (Figure 2B). Serotonin increased Nox1 mRNA expression in control hPASMCs to levels observed in PAH-hPASMCs. In PAH-hPASMCs, serotonin also increased mRNA expression of Nox1 (Figure 2B).

### Regulation of Nrf-2 and Antioxidant Systems by Serotonin

Serotonin increased Nrf-2 activity in control hPASMCs; yet, in PAH-hPASMCs, a statistically significant increase in Nrf-2 activity was not observed with serotonin treatment (Figure 2C). Peroxiredoxin is hyperoxidized and inactivated through sulfonylation. Basal levels of hyperoxidized peroxiredoxin in PAH-hPASMCs were elevated compared with control hPASMCs. Serotonin increased protein expression of hyperoxidized peroxiredoxin in control hPASMCs at 1-hour stimulation, and these effects were blocked by 5-HT_1B_R antagonist, SB224289 (Figure 2D).

### Serotonin Influences Redox Signaling in hPASMCs

**Figure 3.**
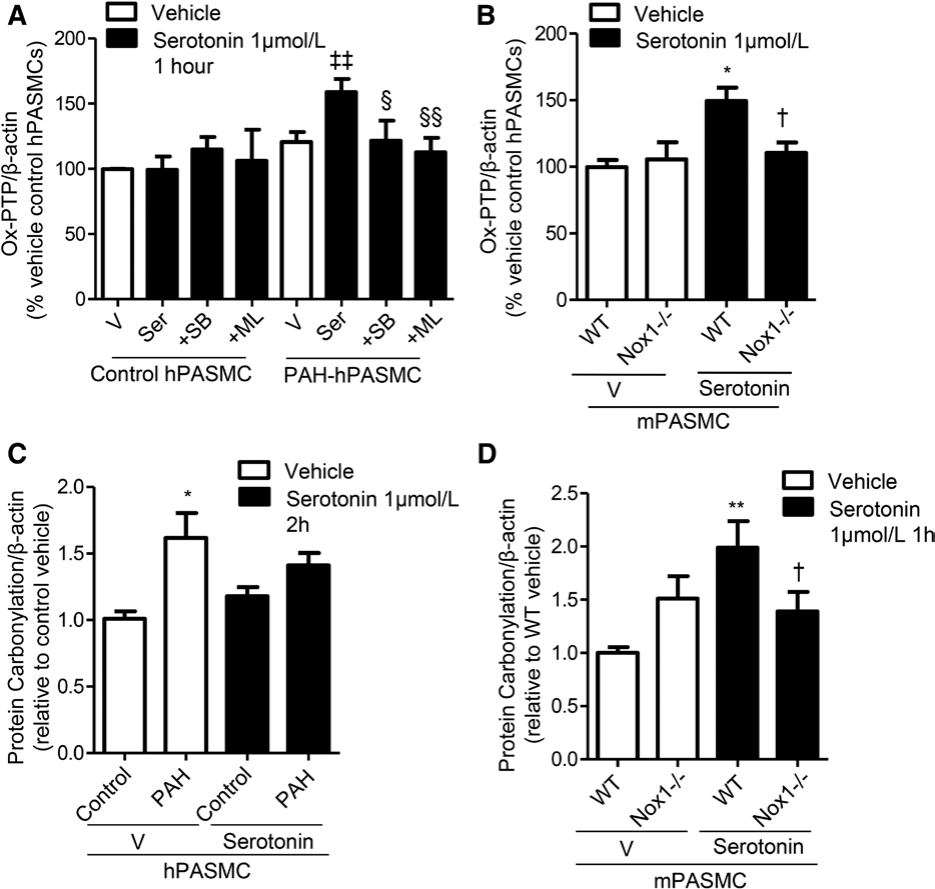
Effects of serotonin on oxidation of protein tyrosine phosphatases (PTPs) and global protein carbonylation. Irreversible oxidation of PTPs using the oxPTP antibody, in response to serotonin in human pulmonary artery smooth muscle cells (hPASMCs) in the presence or absence of SB224289 or Nox1 inhibitor, ML171 (**A**). PTP oxidation in wild-type (WT) and Nox1^−/−^ mouse pulmonary artery smooth muscle cells (mPASMCs) treated with serotonin (**B**). Total protein carbonylation in response to serotonin in hPASMCs (2 h; **C**) and in Nox1^−/−^ mPASMCs (1 h; **D**). Results are representative of 3 to 5 experiments where protein expression is relative to β-actin. *P<0.05, **P<0.01, vs vehicle control hPASMCs or WT vehicle mPASMCs; †*P*<0.05 vs treated WT mPASMCs; ‡‡*P*<0.01 vs vehicle pulmonary arterial hypertension (PAH)-hPASMCs; §*P*<0.05, §§*P*<0.01 vs serotonin-treated human pulmonary artery smooth muscle cells from PAH subjects (PAH-hPASMCs) determined by ANOVA with Tukey post hoc test. ML indicates ML171; SB, SB224289; Ser, serotonin; and V, vehicle.

Serotonin increased irreversible PTP oxidation exclusively in PAH-hPASMCs, inhibited by 5-HT_1B_R inhibitor, SB224289, and the Nox1 inhibitor, ML171 (Figure 3A; Figure IIA in the online-only Data Supplement). Consistent with this, serotonin increased irreversible oxidation of PTPs in WT mPASMCs but had no effect in Nox1^−/−^ mPASMCs (Figure 3B; Figure IIB in the online-only Data Supplement). Carbonylation of proteins is a hallmark of oxidative stress. Basal protein carbonylation was increased in PAH-hPASMCs versus control hPASMCs, with no significant effects of serotonin (Figure 3C). There were increased levels of carbonylation in WT, but not Nox1^−/−^, mPASMCs on serotonin treatment (Figure 3D). Basal Rho-kinase (ROCK) activity was increased in PAH-hPASMCs. Serotonin increased activity in PAH-hPASMCs, an effect that was inhibited by SB224289 and ML171 (Figure III in the online-only Data Supplement).

### Serotonin-Induced Proliferation Involves Nox

**Figure 4.**
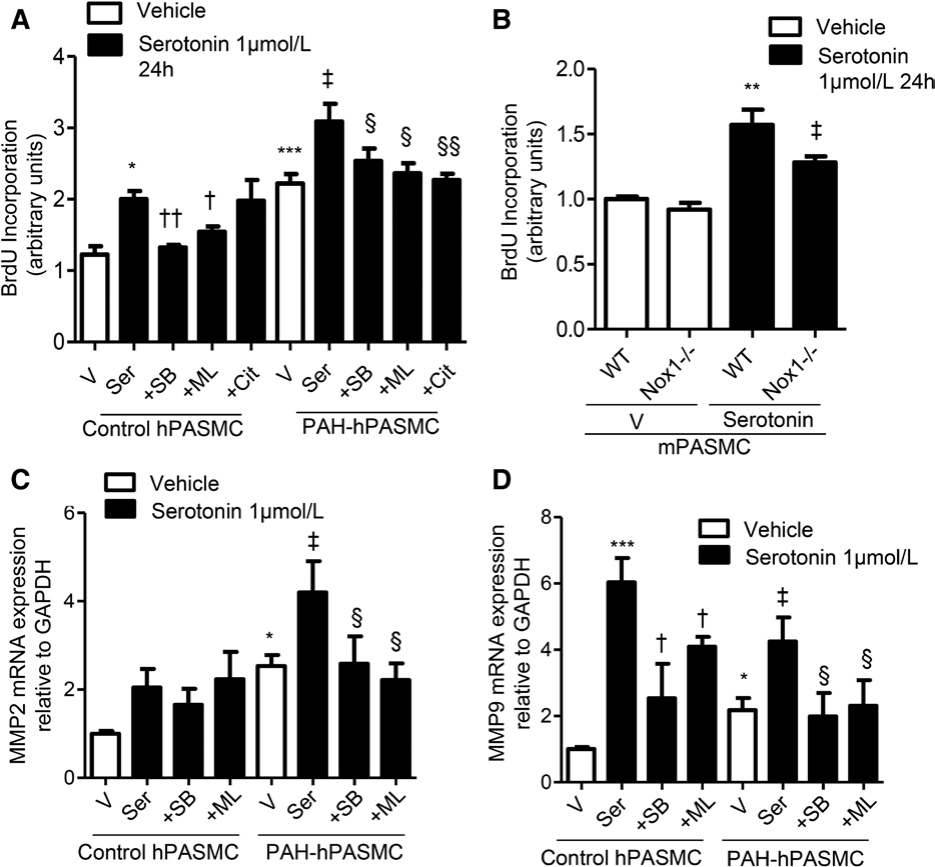
Role of 5-HT_1B_R and Nox1 in serotonin-mediated cell proliferation and extracellular matrix remodeling. To test whether serotonin regulates proliferation in a Nox/reactive oxygen species (ROS)-dependent manner, BrdU (5-Bromo-2′-deoxyuridine) incorporation was assessed in human pulmonary artery smooth muscle cells (hPASMCs; **A**) and wild-type (WT) and Nox1^−/−^ mouse pulmonary artery smooth muscle cells (mPASMCs) in response to serotonin (**B**). Transcript expression of MMP2 (**C**) and MMP9 (**D**) after treatment with serotonin with or without SB224289 or ML171. Results are representative of 5 experiments per group where mRNA expression is normalized to GAPDH. *P<0.05, **P<0.01, ***P<0.001 vs vehicle control hPASMCs or WT vehicle mPASMCs; †*P*<0.05, ††*P*<0.01 vs serotonin-treated control hPASMCs; ‡*P*<0.05 vs vehicle pulmonary arterial hypertension (PAH)-hPASMCs or serotonin-treated mPASMCs; §*P*<0.05, §§*P*<0.01 vs serotonin-treated PAH-hPASMCs determined by ANOVA with Tukey post hoc test. Cit indicates citalopram; ML, ML171; MMP, matrix metalloproteinase; SB, SB224289; Ser, serotonin; and V, vehicle.

Serotonin-induced proliferation was increased in PAH-hPASMCs versus control hPASMCs (Figure 4A). In line with ROS production, these effects were attenuated by the 5-HT_1B_R inhibitor and the Nox1 inhibitor in hPASMCs, with additional inhibitory effects of SERT inhibitor, citalopram, in PAH-hPASMCs (Figure 4A). Consistent with this, proliferation was reduced in Nox1^−/−^ mouse-derived mPASMCs compared with WT mPASMCs when treated with serotonin (Figure 4B).

### Serotonin-Induced Alterations in Markers of Fibrosis and Extracellular Matrix Remodeling Involve Nox1-Derived ROS

Extracellular matrix remodeling is a key process involved in the pathobiology of PAH.^23,24^ Loss of balance between matrix metalloproteinases (MMPs) and TIMPs (tissue inhibitor of matrix metalloproteinases) initiates extracellular matrix and vascular remodeling. Basal MMP2 mRNA was elevated in PAH-hPASMCs and further increased by serotonin; these effects were 5-HT_1B_R- and Nox1-dependent, through the use of pharmacological inhibitors, SB224289 and ML171, respectively (Figure 4C). Basal MMP9 transcript was elevated in PAH-hPASMCs, and serotonin further stimulated MMP9 in both control and PAH-hPASMCs, effects attenuated in the presence of 5-HT_1B_R antagonist, SB224289, and Nox1 inhibitor, ML171 (Figure 4D). Serotonin had no effect on secretion of platelet-derived growth factor subunit B (homodimer) from hPASMCs (Figure IVA in the online-only Data Supplement). Protein expression of platelet-derived growth factor receptor beta (PDGFR-β) was increased under basal conditions in PAH-hPASMCs versus control hPASMCs, whereas in control hPASMCs serotonin increased expression of PDGFR-β where this increase was attenuated in the presence of 5-HT_1B_R antagonist SB224289 (Figure IVB in the online-only Data Supplement).

### 5-HT_1B_R and Nox1 Staining in Human Lung Tissue Sections

In human lung sections from control subjects, 5-HT_1B_R staining was absent. In contrast, 5-HT_1B_R staining was evident in both the endothelium and the smooth muscle of pulmonary arteries in human lung sections from patients with PAH. Nox1 staining was present in smooth muscle of pulmonary arteries of human lung sections derived from both controls and patients with PAH (Figure V in the online-only Data Supplement).

### 5-HT_1B_R Antagonism In Vivo Attenuates Development of PH in the SERT^+^ Mouse and Chronic Hypoxic Mouse

**Figure 5.**
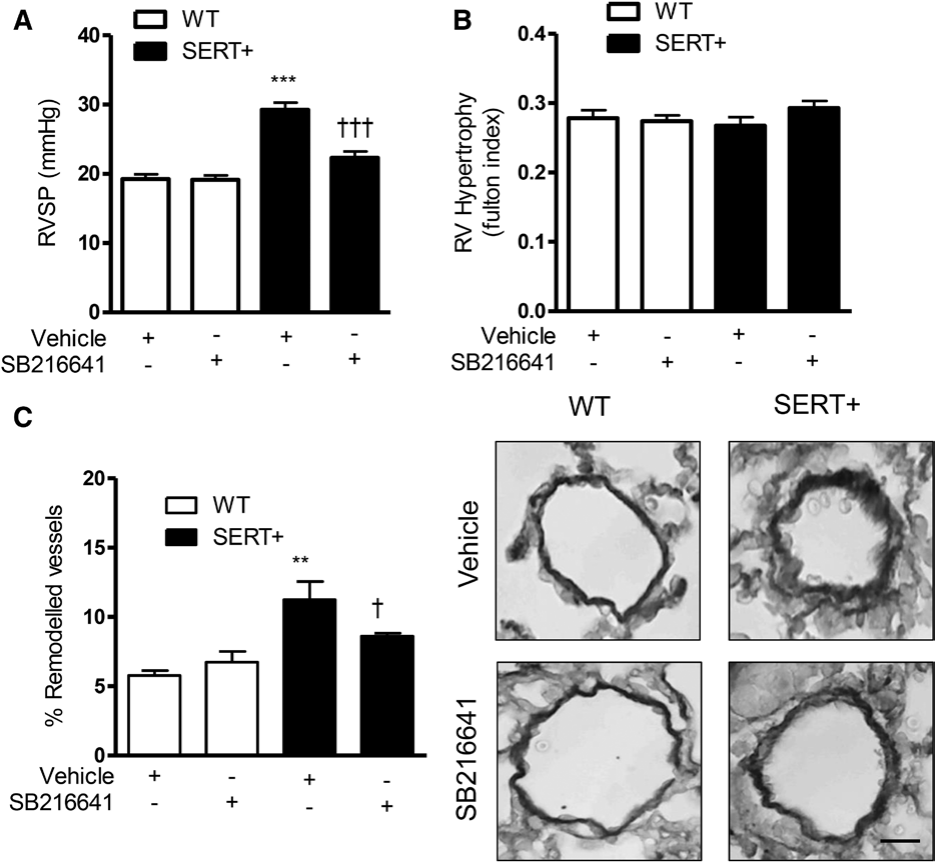
Hemodynamic assessment of pulmonary hypertension in female wild-type (WT) and serotonin transporter overexpressing (SERT^+^) mice treated with SB216641. Right ventricular systolic pressure (RVSP; **A**), right ventricular (RV) hypertrophy (**B**). Effects of SB216641 on percentage of pulmonary vascular remodeling in distal pulmonary arteries in female WT and SERT^+^ mice with representative images (**right**) of pulmonary arteries (Elastin Van Gieson stain; scale bars=50 µm; **C**). Results are mean±SEM, n=8 to 10 per group. ***P<0.001 vs WT vehicle, †††*P*<0.001 vs SERT^+^ vehicle, determined by 2-way ANOVA with Tukey post hoc test. Data are mean±SEM. n=8 to 10 per group. **P<0.01 vs WT vehicle; †*P*<0.05 vs SERT^+^ vehicle, determined by 2-way ANOVA with Tukey post hoc test.

Female mice that overexpress the gene for human SERT demonstrate PH.^6^ Lungs from serotonin transporter overexpressing (SERT^+^) mice demonstrated increased oxidative stress.^25^ The 5-HT_1B_R antagonist, SB216641, reduced the increase in right ventricular systolic pressure (RVSP) in SERT^+^ mice (Figure 5A), and as shown before,^6^ SERT^+^ mice show no change in right ventricular hypertrophy (RVH; Figure 5B). PA remodeling was increased in SERT^+^ mice, and this was reduced by SB216641 (Figure 5C). There was no change in mean arterial pressure (Figure VIA in the online-only Data Supplement) or heart rate (Figure VIB in the online-only Data Supplement) by SB216641 in WT or SERT^+^ mice. In addition, oral administration of SB216641 (15 mg/kg/d) protected against elevations in RVSP, RVH, and the development of pulmonary vascular lesions/remodeling in 8- to 10-week-old female C57BL/6J mice exposed to 15 days chronic hypoxia. Age-matched female mice maintained under normoxic conditions were used as controls (Figure VII in the online-only Data Supplement). PA remodeling was also increased in hypoxic mice compared with their normoxic counterparts, and this remodeling was ameliorated by SB216641 (Figure VIII in the online-only Data Supplement). No effects were observed in normoxic control mice treated with SB216641 (Figures VII and VIII in the online-only Data Supplement).

We investigated whether oxidative stress was regulated by serotonin (and the 5-HT_1B_R) in SERT^+^ mice by immunohistochemical analysis of 8-OHG (8-hydroxyguanosine), a fluorescent ROS probe, which specifically detects oxidative damage to nucleic acids. Female SERT^+^ mice had increased levels of 8-OHG compared with WT mice, and SB21664 treatment reduced 8-OHG levels (Figure IX in the online-only Data Supplement). 8-OHG staining was observed in both the cytoplasm and the nucleus, indicating increased RNA and DNA oxidation.

## Discussion

Our novel findings demonstrate that in hPASMCs serotonin stimulates ROS through Nox, downregulates protective antioxidant mechanisms, stimulates redox signaling and irreversible protein oxidation and carbonylation, and promotes molecular and cellular processes associated with extracellular matrix remodeling and cell growth. These serotonin effects are amplified in hPASMCs from patients with PAH and involve c-Src–regulated Nox1 and 5-HT_1B_R and Nrf-2 dysfunction (summarized in Figure X in the online-only Data Supplement). Using SERT^+^ mice, we confirm, in vivo, our cell-based findings.

The 5-HT_1B_R receptor is highly expressed in human PAs, with increased expression in PAH patients,^4^ and mediates serotonin-induced vasoconstriction and PA remodeling. The 5-HT_2A_R mediates these effects systemically and so the 5-HT_1B_R effects are pulmonary specific, making it a favorable target for PAH. Serotonin-dependent ROS production in hPASMCs was dependent on 5-HT_1B_R in control hPASMCs, whereas in PAH-hPASMCs this was mediated by both the SERT and the 5-HT_1B_R. Consistently, there is an increase in SERT expression in PAs and hPASMCs from PAH patients.^26^ Female mice overexpressing SERT have a PH phenotype.^6^ There is co-operation between SERT and 5-HT_1B_R in human and experimental PAH,^5,20^ and dual blockade of the 5-HT_1B_R and SERT is a plausible therapeutic approach.

Growing evidence implicates a role for Noxs, particularly Nox1 and Nox4, in the pathogenesis of experimental and human PAH.^12,27^ In addition to the constitutive expression of Nox1 in a variety of tissues, Nox1 expression is increased after inflammation, growth factor stimulation, and hypoxia.^28^ Nox1 also plays a critical role in physiological turnover of PASMCs by regulation of intracellular potassium.^29^

We observed that serotonin induces ROS production through Nox1 activation in hPASMCs. Although peak ROS levels induced by serotonin are similar between control and PAH-hPASMCs, we suggest that PAH-hPASMCs are already primed to ROS, as basal levels are increased in PAH-hPASMCs versus control hPASMCs. We note that although ROS levels were sustained in control hPASMCs, they were quickly recovered in PAH-hPASMCs. Although glutathione transcript level is reduced in PAH versus control hPASMCs, there is no further impairment after serotonin stimulation. Similarly, although catalase activity is downregulated by serotonin in control hPASMCs up to 2 hours, in PAH-hPASMCs, catalase activity recovers more rapidly than in control hPASMCs. Moreover, we found that this effect is mediated via c-Src because PP2, which inhibits Src family kinases, attenuated serotonin-induced Nox1 activation. c-Src plays an important role in activation of MAPKs associated with cell growth, apoptosis, and collagen deposition.^30^ Previous studies demonstrated that c-Src is both a redox-sensitive downstream target of Nox and an upstream signaling molecule for Nox activation^31^ and can increase protein abundance subunits and adaptor proteins required for regulation of Nox activity.^16^ Importantly, PP2 reduced serotonin-stimulated ROS formation exclusively in PAH-hPASMCs, suggesting a role for c-Src in serotonin-mediated Nox activation in PAH.

High concentrations of ROS trigger the oxidation of PTPs, resulting in their loss of function as a phosphate acceptor.^32^ In association with excessive ROS production by serotonin, we found an increase in irreversibly oxidized PTPs. Our findings suggest that PTP inhibition may play a role in PAH and that serotonin-induced Nox1-derived ROS may be important in this process.

Other molecular processes that are redox-sensitive relate to total protein carbonyl content, an important index of whole cell protein oxidation.^33^ Accumulation of protein carbonyls has been observed in several human pathologies, including diseases of the lung.^34^ We found increased protein carbonylation in basal conditions, in PAH-hPASMCs compared with control cells, along with increased carbonyl content in response to serotonin in WT mPASMCs, an effect that was absent in mPASMCs from Nox1^−/−^ mice. This suggests a role for Nox1-derived ROS in the regulation of protein carbonylation in hPASMCs. It has been reported that an alternative strategy to antioxidant intervention is to detoxify oxidative-derived carbonyl reaction products.^35^ Thus, strategies to regulate carbonyl content may have clinical value.

Peroxiredoxin is an antioxidant enzyme important in the degradation of H_2_O_2_, which can modulate various receptor signaling pathways and protects cells from oxidation-induced death. The active site cysteine (Cys) of peroxiredoxin is oxidized to the sulfenic acid intermediate (Cys-SOH) when a peroxide is reduced. Yet, because Cys-SOH is unstable, it forms a disulfide with Cys-SH. However, under oxidative stress conditions, the sulfenic intermediate (Cys-SOH) can be easily overoxidized to cysteine sulfinic or sulfonic (Cys-SO_2_H/-SO_3_H) acids, referred to as hyperoxidation. H_2_O_2_ is a common substrate for the peroxidation reaction of peroxiredoxins and also a well-known cause for their hyperoxidation.^22^ In our study, serotonin caused excessive production of H_2_O_2_ in PAH-hPASMCs, as well as decreased activity of H_2_O_2_ reducing enzymes, catalase, and glutathione, in PAH-hPASMCs versus control hPASMCs. These effects were further reduced in serotonin-treated PAH-hPASMCs, indicative of increased production and accumulation of H_2_O_2_ in PAH, potentiated by serotonin. In control hPASMCs, serotonin stimulated ROS production and increased Nrf-2 activity, perhaps as a compensatory mechanism to maintain redox balance. However, in PAH-hPASMCs, serotonin increased ROS production, yet did not induce activity of Nrf-2 as an antioxidant defense mechanism, suggesting overall ROS accumulation and oxidative damage. Of clinical significance, Nrf-2 activators are in human trials for PAH and can attenuate experimental PH.^15^

To better understand the functional significance of serotonin-induced oxidative stress, we studied effects on molecular regulators of vascular contraction and proliferation, hallmarks of vascular remodeling in PAH. In human PAH, ROCK inhibitors cause modest reductions in pulmonary vascular resistance.^36^ As such, ROCK has been implicated in both PA contraction and remodeling.^37^ Serotonin-induced PASMC proliferation, contraction, and migration involve ERK1/2 MAPK and ROCK. Serotonin-mediated signaling of ROCK occurs through 5-HT_1B_R activation in a lung fibroblast cell line.^8^ Here, we show in hPASMCs that serotonin-mediated activation of ROCK is mediated via the 5-HT_1B_R and Nox1-derived ROS. Serotonin stimulated cell proliferation, which was exaggerated in PAH-hPASMCs and dependent on the 5-HT_1B_R and Nox1 because these effects were absent in the presence of inhibitors of this receptor and oxidase, and in Nox1^−/−^ mice. Apoptosis is another key factor in vascular remodeling characteristic of PAH, as such, it has been previously shown that serotonin inhibits PASMC apoptosis through 5-HT_1B_R or SERT.^38^ It would be interesting to understand the role of serotonin and the 5-HT_1B_R in other vascular cell components, such as human PA endothelial cells, primary cultures of such cells are not readily available from control subjects and patients with PAH, and therefore are difficult to study in relation to the human disease.

Structural remodeling of pulmonary arteries through smooth muscle cell migration, proliferation, and oversecretion of extracellular matrix are the most notable pathological changes in PAH.^39^ The MMPs, particularly gelatinases MMP2 and MMP9, are involved in extracellular matrix turnover and hence, in smooth muscle cell migration and proliferation. Of note, promoters of functionally related MMPs, such as MMP2/MMP9, are clearly distinct, pointing to different mechanisms of activation.^40^ As evidenced here and as shown previously, concomitant increments in both MMPs are not always observed. For example, in a rat model of pancreatitis-induced lung injury, MMP9 increased in lung tissue, whereas MMP2 remained unchanged.^41^ However, the promotor for MMP2 does not harbor a TATA box, and expectedly, transcription from these promoters starts at multiple sites. Such that, expression of MMP2 is mainly determined by the ubiquitous Sp1 family of transcription factors, where expression of MMP2 in the main part is constitutive, with only modest sensitivity to induction by growth factors or cytokines.^42^ This is in line with our data showing that MMP2 transcript is induced by serotonin solely in PAH-hPASMCs, whereas MMP9 mRNA expression is sensitive to serotonin stimulation in both control and PAH-hPASMCs. Vascular remodeling in both experimental and human PAH has been shown to involve increased expression of profibrotic proteins. In the lung of PAH patients, accelerated turnover of the extracellular matrix because of increased MMP activity occurs.^23^ Loss of balance between MMPs and TIMPs initiates extracellular matrix and vascular remodeling and is involved in a variety of pathologies.^43^ Serotonin increased expression of the profibrotic signaling proteins MMP2 and MMP9 in hPASMCs. These effects were absent in hPASMCs pretreated with inhibitors of the 5-HT_1B_R or Nox1, suggesting an important role for Nox1 in the profibrotic effects of serotonin.

The rationale for platelet-derived growth factor involvement in the pathogenesis of PAH is strong, with increased expression of ligand and phosphorylated receptor in patient lung tissue.^44^ However, we did not observe any change in the platelet-derived growth factor subunit B (homodimer) levels in the spent culture medium from serotonin-stimulated control and PAH-hPASMCs. Because both serotonin and PDGFR-β have been associated with clinical and experimental PH, and patients with PA hypertension have enhanced activation of PDGFR-β in their lungs,^44^ we investigated whether there is a relationship between these molecules in hPASMCs. Expression of PDGFR-β was increased under basal conditions in PAH-hPASMCs versus control hPASMCs, whereas in control hPASMCs, serotonin increased expression of PDGFR-β, where this increase was absent in the presence of 5-HT_1B_R antagonist SB224289. A previous study in bovine-derived PASMCs observed serotonin-stimulated production of ROS serving as an intermediate in the transactivation of PDGFR-β by serotonin, where oxidation of PTPs was suggested as the mechanism involving SERT rather than the 5-HT receptors.^45^ In line, we observed that in female human PASMCs, PDGFR-β expression is increased in PAH-hPASMCs versus control hPASMCs. In control hPASMCs, expression of PDGFR-β was elevated by serotonin in a 5-HT_1B_R–dependent manner. This is consistent with recognized cross talk between platelet-derived growth factor and serotonin signaling in pulmonary arteries.^46^

To test the pathophysiological significance of our findings, we performed in vivo studies assessing the effects of the 5-HT_1B_R antagonist, SB216641, in 2 mouse models of PH, the chronic hypoxic mouse, and in a serotonin-dependent mouse model of PH, the SERT^+^ mouse. Female C57BL6/J mice exposed to hypoxic conditions developed elevated RVSP, RVH, and evidence of pulmonary vascular remodeling parameters, which were prevented in those mice treated with the 5-HT_1B_R antagonist, SB216641. Similarly, female SERT^+^ mice developed increased RVSP and PA remodeling at 20 weeks of age; protective effects of SB216641 treatment was observed. RVH remained unaffected, in line with previous work where our group and others have shown a dissociation between RVSP and RVH in the SERT^+^ mouse.^6^ We previously demonstrated increased ROS expression in the SERT^+^ mouse lung^25^; lung ROS was reduced by the 5-HT_1B_R antagonist, suggesting that the 5-HT_1B_R/ROS axis is important both in vitro in cultured PASMCs and in vivo in the mouse lung. In addition, in support of the role of 5-HT_1B_R in human PAH, we observed marked 5-HT_1B_R staining in the endothelium and smooth muscle cell layers of the vasculature in human lung sections from PAH subjects, staining that was absent in lung sections from control subjects.

In conclusion, we show for the first time in hPASMCs from PAH patients that serotonin increases Nox1-dependent ROS generation and decreases Nrf-2-antioxidant systems through c-Src–dependent processes that contribute to oxidation of proteins, DNA damage, and redox-sensitive proliferation and fibrosis of hPASMCs, processes involved in PAH. In line, we observed ROS-dependent protective effects of 5-HT_1B_R antagonism in the SERT^+^ mouse model of PH. We identify the 5-HT_1B_R and both 5-HT_1B_R and SERT in PAH-derived PASMCs, as being particularly important in Nox1-derived ROS production and in serotonin-mediated vascular effects in PAH. Our study provides new molecular insights through c-Src/Nox1/ROS and Nrf-2 antioxidant mechanisms, whereby serotonin influences hPASMC function, which when upregulated may contribute to vascular injury in PAH (summarized in Figure X in the online-only Data Supplement).

## Acknowledgments

We are grateful to Margaret Nilsen for the technical support and Professor N.W. Morrell (University of Cambridge, UK) for the supply of human pulmonary artery smooth muscle cells.

## Sources of Funding

This work was funded by a Biotechnology and Biological Sciences Research Council DTP studentship (2012/168760-01) and the British Heart Foundation (RG/11/7/28916; CH/12/4/29762, RG/13/7/30099). This article was part-funded by grant RE/13/5/30177 from the British Heart Foundation Centre of Research Excellence award.

## Disclosures

None.

## Supplementary Material

## Notes

Highlights

- Serotonin-induced proliferation of vascular cells, via the serotonin transporter and the 5-HT_1B_ receptor, has been associated with the development of pulmonary arterial hypertension.
- Serotonin stimulates production of reactive oxygen species but mechanisms underlying this, and the significance of serotonin-induced reactive oxygen species in the development of pulmonary arterial hypertension, remain unclear.
- Here, we show that redox-sensitive effects of serotonin are associated with Nrf-2 dysregulation, contributing to oxidative stress, protein oxidation, proliferation, and extracellular matrix remodeling in pulmonary artery smooth muscle cells from pulmonary arterial hypertension patients.
- We demonstrate in vivo that this may contribute to the pathobiology of pulmonary hypertension.
- Therefore, inhibition of the serotonin-mediated redox system or activation of Nrf-2 could be novel therapeutic strategies in pulmonary arterial hypertension.

## References

[1] MacLean MR, Dempsie Y (2009). Serotonin and pulmonary hypertension—from bench to bedside?. Curr Opin Pharmacol.

[2] Fanburg BL, Lee SL (1997). A new role for an old molecule: serotonin as a mitogen.. Am J Physiol.

[3] MacLean MR (2007). Pulmonary hypertension and the serotonin hypothesis: where are we now?. Int J Clin Pract Suppl.

[4] Morecroft I, Heeley RP, Prentice HM, Kirk A, MacLean MR (1999). 5-hydroxytryptamine receptors mediating contraction in human small muscular pulmonary arteries: importance of the 5-HT1B receptor.. Br J Pharmacol.

[5] Lawrie A, Spiekerkoetter E, Martinez EC, Ambartsumian N, Sheward WJ, MacLean MR, Harmar AJ, Schmidt AM, Lukanidin E, Rabinovitch M (2005). Interdependent serotonin transporter and receptor pathways regulate S100A4/Mts1, a gene associated with pulmonary vascular disease.. Circ Res.

[6] MacLean MR, Deuchar GA, Hicks MN, Morecroft I, Shen S, Sheward J, Colston J, Loughlin L, Nilsen M, Dempsie Y, Harmar A (2004). Overexpression of the 5-hydroxytryptamine transporter gene: effect on pulmonary hemodynamics and hypoxia-induced pulmonary hypertension.. Circulation.

[7] Morecroft I, Loughlin L, Nilsen M, Colston J, Dempsie Y, Sheward J, Harmar A, MacLean MR (2005). Functional interactions between 5-hydroxytryptamine receptors and the serotonin transporter in pulmonary arteries.. J Pharmacol Exp Ther.

[8] Mair KM, MacLean MR, Morecroft I, Dempsie Y, Palmer TM (2008). Novel interactions between the 5-HT transporter, 5-HT1B receptors and Rho kinase *in vivo* and in pulmonary fibroblasts.. Br J Pharmacol.

[9] MacLean MR, Morecroft I (2001). Increased contractile response to 5-hydroxytryptamine1-receptor stimulation in pulmonary arteries from chronic hypoxic rats: role of pharmacological synergy.. Br J Pharmacol.

[10] Bedard K, Krause KH (2007). The NOX family of ROS-generating NADPH oxidases: physiology and pathophysiology.. Physiol Rev.

[11] Tabima DM, Frizzell S, Gladwin MT (2012). Reactive oxygen and nitrogen species in pulmonary hypertension.. Free Radic Biol Med.

[12] Hood KY, Montezano AC, Harvey AP, Nilsen M, MacLean MR, Touyz RM (2016). Nicotinamide adenine dinucleotide phosphate oxidase-mediated redox signaling and vascular remodeling by 16α-hydroxyestrone in human pulmonary artery cells: implications in pulmonary arterial hypertension.. Hypertension.

[13] Dalle-Donne I, Rossi R, Giustarini D, Milzani A, Colombo R (2003). Protein carbonyl groups as biomarkers of oxidative stress.. Clin Chim Acta.

[14] Briones AM, Touyz RM (2010). Oxidative stress and hypertension: current concepts.. Curr Hypertens Rep.

[15] Eba S, Hoshikawa Y, Moriguchi T, Mitsuishi Y, Satoh H, Ishida K, Watanabe T, Shimizu T, Shimokawa H, Okada Y, Yamamoto M, Kondo T (2013). The nuclear factor erythroid 2-related factor 2 activator oltipraz attenuates chronic hypoxia-induced cardiopulmonary alterations in mice.. Am J Respir Cell Mol Biol.

[16] Touyz RM, Yao G, Schiffrin EL (2003). c-Src induces phosphorylation and translocation of p47phox: role in superoxide generation by angiotensin II in human vascular smooth muscle cells.. Arterioscler Thromb Vasc Biol.

[17] Pullamsetti SS, Berghausen EM, Dabral S, Tretyn A, Butrous E, Savai R, Butrous G, Dahal BK, Brandes RP, Ghofrani HA, Weissmann N, Grimminger F, Seeger W, Rosenkranz S, Schermuly RT (2012). Role of Src tyrosine kinases in experimental pulmonary hypertension.. Arterioscler Thromb Vasc Biol.

[18] Lee SL, Wang WW, Fanburg BL (1998). Superoxide as an intermediate signal for serotonin-induced mitogenesis.. Free Radic Biol Med.

[19] Liu JQ, Folz RJ (2004). Extracellular superoxide enhances 5-HT-induced murine pulmonary artery vasoconstriction.. Am J Physiol Lung Cell Mol Physiol.

[20] Morecroft I, Pang L, Baranowska M, Nilsen M, Loughlin L, Dempsie Y, Millet C, MacLean MR (2010). *In vivo* effects of a combined 5-HT1B receptor/SERT antagonist in experimental pulmonary hypertension.. Cardiovasc Res.

[21] Callera GE, Touyz RM, Tostes RC, Yogi A, He Y, Malkinson S, Schiffrin EL (2005). Aldosterone activates vascular p38MAP kinase and NADPH oxidase via c-Src.. Hypertension.

[22] Lim JC, Choi HI, Park YS, Nam HW, Woo HA, Kwon KS, Kim YS, Rhee SG, Kim K, Chae HZ (2008). Irreversible oxidation of the active-site cysteine of peroxiredoxin to cysteine sulfonic acid for enhanced molecular chaperone activity.. J Biol Chem.

[23] Wei L, Warburton RR, Preston IR, Roberts KE, Comhair SA, Erzurum SC, Hill NS, Fanburg BL (2012). Serotonylated fibronectin is elevated in pulmonary hypertension.. Am J Physiol Lung Cell Mol Physiol.

[24] Rabinovitch M (2001). Pathobiology of pulmonary hypertension. Extracellular matrix.. Clin Chest Med.

[25] Johansen AK, Dean A, Morecroft I, Hood K, Nilsen M, Loughlin L, Anagnostopoulou A, Touyz RM, White K, MacLean MR (2016). The serotonin transporter promotes a pathological estrogen metabolic pathway in pulmonary hypertension via cytochrome P450 1B1.. Pulm Circ.

[26] Eddahibi S, Humbert M, Fadel E, Raffestin B, Darmon M, Capron F, Simonneau G, Dartevelle P, Hamon M, Adnot S (2001). Serotonin transporter overexpression is responsible for pulmonary artery smooth muscle hyperplasia in primary pulmonary hypertension.. J Clin Invest.

[27] Mittal M, Roth M, König P (2007). Hypoxia-dependent regulation of nonphagocytic NADPH oxidase subunit NOX4 in the pulmonary vasculature.. Circ Res.

[28] Fu XJ, Peng YB, Hu YP, Shi YZ, Yao M, Zhang X (2014). NADPH oxidase 1 and its derived reactive oxygen species mediated tissue injury and repair.. Oxid Med Cell Longev.

[29] Iwata K, Ikami K, Matsuno K (2014). Deficiency of NOX1/nicotinamide adenine dinucleotide phosphate, reduced form oxidase leads to pulmonary vascular remodeling.. Arterioscler Thromb Vasc Biol.

[30] Touyz RM (2003). Recent advances in intracellular signalling in hypertension.. Curr Opin Nephrol Hypertens.

[31] Yang S, Hardaway M, Sun G, Ries WL, Key LL (2000). Superoxide generation and tyrosine kinase.. Biochem Cell Biol.

[32] Frijhoff J, Dagnell M, Godfrey R, Ostman A (2014). Regulation of protein tyrosine phosphatase oxidation in cell adhesion and migration.. Antioxid Redox Signal.

[33] Chevion M, Berenshtein E, Stadtman ER (2000). Human studies related to protein oxidation: protein carbonyl content as a marker of damage.. Free Radic Res.

[34] Dalle-Donne I, Giustarini D, Colombo R, Rossi R, Milzani A (2003). Protein carbonylation in human diseases.. Trends Mol Med.

[35] Negre-Salvayre A, Coatrieux C, Ingueneau C, Salvayre R (2008). Advanced lipid peroxidation end products in oxidative damage to proteins. Potential role in diseases and therapeutic prospects for the inhibitors.. Br J Pharmacol.

[36] Oka M, Homma N, Taraseviciene-Stewart L, Morris KG, Kraskauskas D, Burns N, Voelkel NF, McMurtry IF (2007). Rho kinase-mediated vasoconstriction is important in severe occlusive pulmonary arterial hypertension in rats.. Circ Res.

[37] Fukumoto Y, Tawara S, Shimokawa H (2007). Recent progress in the treatment of pulmonary arterial hypertension: expectation for rho-kinase inhibitors.. Tohoku J Exp Med.

[38] Liu Y, Tian HY, Yan XL, Fan FL, Wang WP, Han JL, Zhang JB, Ma Q, Meng Y, Wei F (2013). Serotonin inhibits apoptosis of pulmonary artery smooth muscle cell by pERK1/2 and PDK through 5-HT1B receptors and 5-HT transporters.. Cardiovasc Pathol.

[39] Case D, Irwin D, Ivester C (2007). Mice deficient in galectin-1 exhibit attenuated physiological responses to chronic hypoxia-induced pulmonary hypertension.. Am J Physiol Lung Cell Mol Physiol.

[40] Yan C, Boyd DD (2007). Regulation of matrix metalloproteinase gene expression.. J Cell Physiol.

[41] Keck T, Balcom JH, Fernández-del Castillo C, Antoniu BA, Warshaw AL (2002). Matrix metalloproteinase-9 promotes neutrophil migration and alveolar capillary leakage in pancreatitis-associated lung injury in the rat.. Gastroenterology.

[42] Chakraborti S, Mandal M, Das S, Mandal A, Chakraborti T (2003). Regulation of matrix metalloproteinases: an overview.. Mol Cell Biochem.

[43] Somerville RP, Oblander SA, Apte SS (2003). Matrix metalloproteinases: old dogs with new tricks.. Genome Biol.

[44] Schermuly RT, Dony E, Ghofrani HA, Pullamsetti S, Savai R, Roth M, Sydykov A, Lai YJ, Weissmann N, Seeger W, Grimminger F (2005). Reversal of experimental pulmonary hypertension by PDGF inhibition.. J Clin Invest.

[45] Liu Y, Li M, Warburton RR, Hill NS, Fanburg BL (2007). The 5-HT transporter transactivates the PDGFbeta receptor in pulmonary artery smooth muscle cells.. FASEB J.

[46] Ciuclan L, Hussey MJ, Burton V (2013). Imatinib attenuates hypoxia-induced pulmonary arterial hypertension pathology via reduction in 5-hydroxytryptamine through inhibition of tryptophan hydroxylase 1 expression.. Am J Respir Crit Care Med.

