# Supplementary figures and images for: Serotonin Signaling Through the 5-HT1B Receptor and NADPH Oxidase 1 in Pulmonary Arterial Hypertension
Source: Arterioscler Thromb Vasc Biol. 2017 Jun 21;37(7):1361–70. doi: 10.1161/ATVBAHA.116.308929 (PMC5478178; doi:10.1161/ATVBAHA.116.308929)

**Pulmonary artery smooth muscle cells**

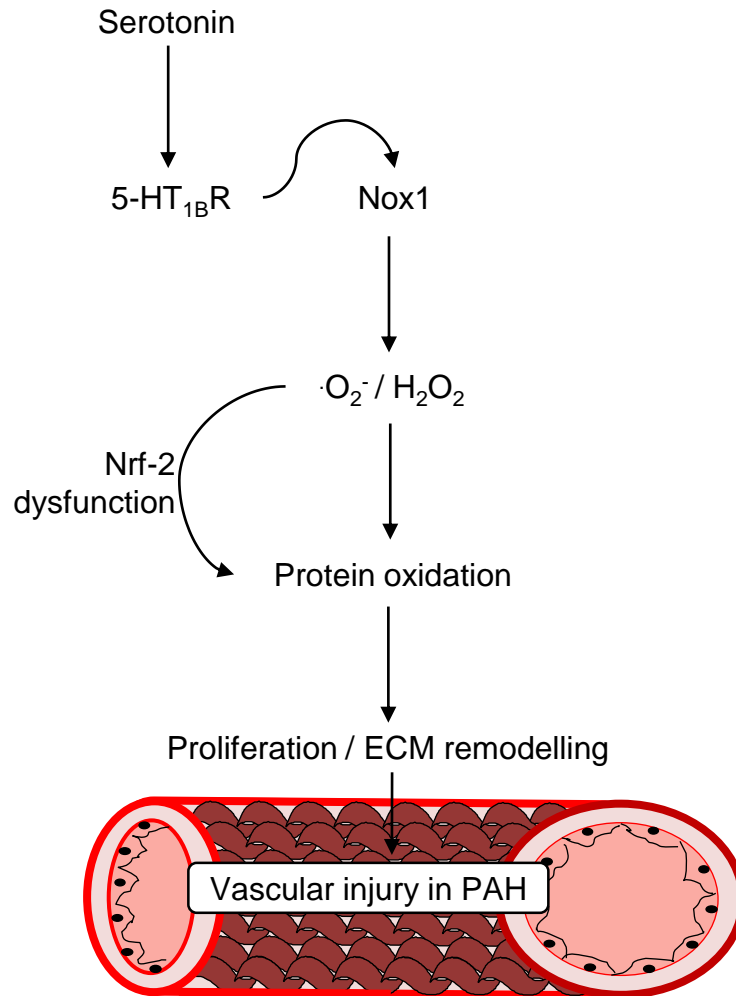

Supplement: Supplementary file 1 [file atv-37-1361-s001.pdf]
