# Supplementary material for: Serotonin Signaling Through the 5-HT1B Receptor and NADPH Oxidase 1 in Pulmonary Arterial Hypertension
Source: Arterioscler Thromb Vasc Biol. 2017 Jun 21;37(7):1361–70. doi: 10.1161/ATVBAHA.116.308929 (PMC5478178; doi:10.1161/ATVBAHA.116.308929)
Supplement: Supplementary file 2 [file atv-37-1361-s002.pdf]

Materials and Methods

Serotonin Signaling Through the 5-HT_{1B} Receptor and NADPH Oxidase 1 in Pulmonary Arterial Hypertension.

Katie Y Hood PhD, Kirsty M Mair PhD, Adam P Harvey PhD, Augusto C Montezano PhD, Rhian M Touyz MD PhD*, Margaret R MacLean PhD*.
Joint senior authors

Institute of Cardiovascular and Medical Sciences, University of Glasgow, United Kingdom.

Cell Culture

In vitro studies were performed using primary hPASMC from distal arteries from PAH patients (PAH-hPASMCs). Cells were explanted as described[1], and were provided by N.W. Morrell, University of Cambridge, UK. PASMCs from non-PAH subjects (control hPASMCs) were also studied (patient details in Supp. Table I) where PAH was not suspected on clinical or radiological grounds in these subjects and cells were derived from macroscopically normal tissue. Experimental procedures using hPASMCs were approved by Cambridgeshire 1 Research Ethics Committee (reference: 08/H0304/56). Additional *in vitro* studies were performed in primary cultured mouse PASMCs (mPASMCs) from the intralobar pulmonary arteries of female Nox1-/- mice and WT littermates (C57BL/6J background), used between passage 2 and 4, as described previously[2].

Cell Protocols

Cells were cultured in Dulbecco's Modified Eagle Medium (DMEM; Gibco, UK) supplemented with antibiotic antimycotic solution (containing 0.25µg/ml amphotericin B; 100U/ml penicillin; 100µg/ml streptomycin; Sigma-Aldrich, UK) and 10% (v/v) fetal bovine serum (Sera Laboratories International, West Sussex, UK). Cells were grown to 70% confluence before serum deprivation for 24 hours in 0.5% fetal bovine serum in phenol-red free DMEM prior to experiments to render them quiescent. Cells were stimulated with serotonin (1µmol/L; 5 minutes-24 hours). In some protocols, cells were pre-treated (30 minutes) with pharmacological inhibitors Acetylphenothiazine (ML171; Nox1 inhibitor, 1µmol/L); 1'-Methyl-5-[[2'-methyl-4'-(5-methyl-1,2,4-oxadiazol-3-yl)biphenyl-4-yl]carbonyl]-2,3,6,7-tetrahydrospiro[furo[2,3-*f*]indole-3,4'-piperidinehydrochloride (SB224289; 5-HT_{1B}R antagonist, 0.1µmol/L); 3-[2-[4-(4-Fluorobenzoyl)-1-piperidinyl]ethyl]-2,4[1*H*,3*H*]-quinazolinedione tartrate (ketanserin; 5-$HT_{2A/1D}$R antagonist, 0.1µmol/L); 1-[3-(dimethylamino)propyl]-1-(4-fluorophenyl)-1,3-dihydro-5-isobenzofurancarbonitrile hydrobromide (citalopram; SERT inhibitor, 0.1µmol/L); 4-Amino-3-(4-chlorophenyl)-1-(t-butyl)-1H-pyrazolo[3,4-d] pyrimidine (PP2; c-Src inhibitor, 0.1µmol/L); 4-Hydroxy-2,2,6,6-tetramethylpiperidine 1-oxyl (tempol; SOD mimetic, 10µmol/L). Doses were based on preliminary experiments and published data.

Reagents and Antibodies

The following antibodies were used: anti-PRDX-SO_2/SO_3H (Abcam, ab16830); anti-PCNA (Santa Cruz, sc-56); anti-Nox1 (Abcam, ab55831); anti-Ox-PTP (R&D systems, MAB2844) and anti-β-actin (Abcam, ab8229). Serotonin (Cat.#3547), PP2, a Src kinase inhibitor (Cat.#P0042) and SB224289, (5-HT_{1B} R antagonist; Cat.#S201); ML171 (Nox1 inhibitor; Cat.#4653); Tempol (SOD mimetic; Cat.#3082); Ketanserin (5-$HT_{2A/1D}$R antagonist; Cat#0908) and Citalopram (SERT inhibitor; Cat#1427) were purchased from Tocris, UK. Doses were based on preliminary experiments and published data.

Lucigenin-Enhanced Chemiluminescence

Lucigenin-enhanced chemiluminescence was used to determine ROS generation as described previously[3]. ROS production was determined in control hPASMC, PAH-hPASMCs and mPASMCs stimulated with serotonin for 5 minutes to 48 hours. Inhibitor studies (30 minutes' pre-incubation) were carried out at the peak time points for ROS production (1 hour). After stimulation, cells were washed with ice-cold PBS and harvested in lysis buffer (20mmol/L KH_2PO_4, 1 mmol/L EGTA, 1µg/mL aprotinin, 1µg/mL leupeptin, 1µg/mL pepstatin, and 1mmol/L PMSF). 50µl of sample was added to a suspension containing 175µl of assay buffer (50mmol/L KH_2PO_4, 1mmol/L EGTA,150mmol/L sucrose) and lucigenin (5µmol/L). NADPH (100µmol/L) was added to the suspension containing lucigenin (5µmol/L). Luminescence was measured with a luminometer (AutoLumat LB 953, Berthold)

before and after stimulation with NADPH. A buffer blank was subtracted from each reading. Superoxide anion production was inhibited by tempol and was expressed as relative luminescence units (RLU)/µg protein, relative to vehicle conditions. For analysis, basal relative light units (RLU) values were subtracted from NADPH-stimulated values. RLU were corrected to sample protein concentration (µg/ml), as assessed by DC assay (Bio-Rad). Results in RLU/ug protein were then normalised to their respective vehicle control hPASMCs data. Data is expressed as relative percentage (%) of vehicle control hPASMCs.

Amplex Red Assays

H_2O_2 production and catalase activity was assessed with Amplex Red Hydrogen Peroxide/Peroxidase Assay, or Amplex Red Catalase Assay, (Thermo Fisher, UK) according to instructions, as described previously[4].

Real-Time PCR

Quantitative real-time PCR was used to analyse mRNA expression using SYBR Green I as previously described[4]. Primers used were designed using the Primer 3 software online (Supp. Table II).

Nuclear factor (erythroid-derived 2)-like 2 (Nrf-2) Activity Assay

Nrf-2 activity was determined with the TransAM Nrf-2 assay (Active Motif, USA) following manufacturer's instructions. Briefly, to assess Nrf-2 nuclear accumulation, samples were prepared according to the manufacturer's protocol using a nuclear extract kit (Active Motif, USA). Nuclear preparations (10µg) were used for the TransAM Nrf2 ELISA kit (Active Motif, USA) to measure DNA binding of activated Nrf2 nuclear protein, as determined by absorbance measurements at 450nm, as described[4].

Measurement of Rho Kinase Activity

Rho kinase (ROCK) activity was assessed using the ROCK Activity Assay (Cell Biolabs Inc) following manufacturer's instructions[5].

Measurement of PDGF-BB levels

PDGF-BB levels in spent medium from control and PAH-hPASMCs, was assessed using the PDGF-BB ELISA (R&D Systems, cat# DBB00) according to manufacturer's instructions.

Immunoblotting

Proteins were extracted from hPASMCs and protein expression of PDGFR-β (Abcam; cat# ab32570) and hyper-oxidized peroxiredoxin (PRDX-SO_3H) was examined by immunoblotting as described previously[6]. All antibodies were used at 1:1000 dilution unless otherwise stated. Washed membranes were incubated with horseradish peroxidase - conjugated secondary antibodies and probed for immunoreactive proteins by chemiluminescence (WestPico, Pierce).

Protein tyrosine phosphatase oxidation

Irreversible oxidation of PTPs was assessed using anti-Ox-PTP antibody that recognizes the sulfonic acid form of PTP cysteine residues. Briefly, irreversible oxidation of protein tyrosine phosphatases (PTPs) was assessed by immunoblotting using an antibody (anti-Ox-PTP; 1:2000 dilution, R&D Systems) that specifically recognizes the sulfonic acid form of PTP cysteine residues. Following treatment, the sample is alkylated using N-ethylmaleimide (NEM; 1mmol/L) to protect reduced cysteines. Subsequently, the samples are treated with dithiothreitol (DTT; 1mmol/L) and pervanadate (100µmol/L) to convert reduced cysteines to

the sulfonic acid form. Finally, the sulfonic acid form is detected using the Ox-PTP antibody by immunoblotting as previously described[7].

OxyBlot

Protein carbonylation was assessed by OxyBlot Protein Oxidation Detection Kit (Millipore) following manufacturer's instructions[8].

5-Bromo-2'-deoxyuridine (BrdU) Incorporation Assay

Proliferation was measured using the BrdU Incorporation assay (Millipore) following manufacturer's instructions as described[6]. Briefly, cells were seeded onto a 96-well plate and starved overnight before stimulation. Cells were incubated with BrdU for 24 hours. Absorbance was obtained at dual wavelength (450nm and 595nm) with a spectrophotometer (Spectra Max; Molecular Devices, Sunnyvale, CA, USA). The results were normalized as percent of control vehicle conditions

Ethical Considerations: SERT+ mice and C57BL/6J mice

The investigation conforms with the UK Animal Procedures Act (1986) and ARRIVE Guidelines. All mice used were 20-week-old females. Mice were housed in controlled environmental conditions with a 12-hour light/dark cycle with free access to food and water.

SERT+ Mice

SERT+ mice (background: C57BL/6JxCBA) have been previously described[9]. 20-week old female SERT+ mice underwent 14-days treatment with 5-HT_{1B}R antagonist (SB216641; 15mg/kg/day, oral gavage) or vehicle (dH_2O). Age-matched littermates were studied as controls.

Chronic Hypoxia Model of Pulmonary Hypertension

Female C57BL/6J mice (aged 8-10 weeks) were exposed to hypoxic conditions for 15 days using a hypobaric chamber. Age-matched normoxic female mice were assessed as controls. From days 1 to 15, mice underwent treatment with either 5-HT_{1B}R antagonist (SB216641; 15mg/kg/day, oral gavage) or vehicle (dH_2O).

***In vivo* Haemodynamic Measurements**

Pressure measurements were conducted and analysed as described previously[10]. Inhalation anaesthesia was used throughout the procedure (1–3% isoflurane/O_2). Depth of anaesthesia was assessed by loss of pain and corneal reflexes. Briefly, right ventricular systolic pressure (RVSP) was measured via a 25-gauge needle advanced into the right ventricle (RV) using a trans-diaphragmatic approach. Mean systemic arterial pressure (mSAP) was obtained via a micro-cannula inserted into the carotid artery. Mice were euthanized by cervical dislocation and heart and lungs removed for subsequent analysis as described below. Eight to 10 mice per group were studied.

Measurement of Right Ventricular Hypertrophy

Right ventricular hypertrophy (RVH) was assessed as weight of the RV free wall/ weight of the left ventricle with the septum.

Human Lung Immunohistochemistry

Formalin-fixed, paraffin embedded tissue sections from human control lung and human PAH lung, were stained using anti-5-HT_{1B}R antibody (Abcam ab13896 1:400), and anti-Nox1 antibody (Sigma, SAB4200097; 1:200). An anti-rabbit IgG secondary antibody was used for

each primary antibody (5µg/ml; Vector Laboratories IMMpress kit) and protein immuno-localisation was visualized with the alkaline phosphatase substrate kit (Vector labs UK; sk-5100).

Lung Immunohistochemistry to assess PA remodeling

At harvest, the lung was perfusion-fixed via the trachea, processed into paraffin where sections (3µm) were cut and stained for elastin and collagen using Millers Elastin and Picro-Sirius red for identification of vascular thickening. Remodeling of pulmonary arteries distal to the airways, was defined by the presence of a double elastic lamina and/or by an increase in the vessel wall diameter in more than 50% of the arterial wall. The total number of remodelled vessels was expressed over the total number of vessels present in a lung section as assessed by a blinded investigator, where at least 100 pulmonary arteries of <80µm external diameter per lung were scored. Lung sections from six mice from each group were studied. Analysis was carried out in a blinded fashion as described previously[11].

Reactive Oxygen Species Determination in Lung Sections by Immunofluorescence

Immunohistochemistry of the ROS marker 8-hydroxyguanosine (8-OHG) was determined in the lungs of wild-type (WT) and SERT+ mice treated with 5-HT_{1B}R antagonist, SB216641, or vehicle, as previously described[12]. Briefly, sections were deparaffinised in a xylene-ethanol-water gradient. Antigen retrieval was performed by boiling slides in EDTA (pH 8.0) for 15 minutes before a 1-hour incubation in a humidified chamber at room temperature with 10% donkey serum/1% bovine serum albumin in 1× TBS-T to block non-specific binding. Sections were incubated with anti- 8-OHG overnight at 4°C (5 µg/mL, ab10802, Abcam). For identification of 8-OHG-positive cells, sections were incubated with Alexa-fluor-488-conjugated donkey anti-goat secondary antibody (A-11055, Molecular Probes, Life Technologies, UK) for 45 minutes at room temperature in the dark. Lipofuscin-mediated auto-fluorescence was removed with 0.1% Sudan Black B (Sigma-Aldrich) in methanol w/v for 10 minutes. Slides were mounted with Pro-Long Gold anti-fade mounting media containing DAPI (4′,6-diamidino-2-phenylindole; P-36931, Molecular Probes, Life Technologies). Fluorescence imaging was measured in an Axiovert 200M microscope with a laser-scanning module LSM 510 (Carl Zeiss, Germany). DAPI was excited at 405nm and Alexa-Fluor 488 at 488nm. Images were captured with the LSM 510 Zen evaluation software (Zeiss, UK).

Statistical Analysis

Mean values ± SEM were calculated and statistical comparisons made using Graph Pad Prism 7.02 software, with 1-way or 2-way ANOVA followed by Tukey’s post hoc test or two-tailed Student’s-*t*-test where appropriate. $P<0.05$ was considered statistically significant (*).

References

1. Morrell NW, Upton PD, Kotecha S, Huntley A, Yacoub MH, Polak JM, Wharton J. Angiotensin II activates mapk and stimulates growth of human pulmonary artery smooth muscle via at1 receptors. *Am J Physiol*. 1999;277:L440-448.
2. Long L, MacLean MR, Jeffery TK, Morecroft I, Yang X, Rudarakanchana N, Southwood M, James V, Trembath RC, Morrell NW. Serotonin increases susceptibility to pulmonary hypertension in bmpr2-deficient mice. *Circ Res*. 2006;98:818-827.
3. Touyz R, Yao G, Viel E, Amiri F, Schiffrin E. Angiotensin II and endothelin-1 regulate map kinases through different redox-dependent mechanisms in human vascular smooth muscle cells. *Journal of Hypertension*. 2004;22:1141-1149.
4. Lopes RA, Neves KB, Tostes RC, Montezano AC, Touyz RM. Downregulation of nuclear factor erythroid 2-related factor and associated antioxidant genes contributes to redox-sensitive vascular dysfunction in hypertension. *Hypertension*. 2015;66:1240-1250.
5. Cat AND, Antunes TT, Callera GE, Sanchez A, Tsiropoulou S, Dulak-Lis MG, Anagnostopoulou A, He Y, Montezano AC, Jaisser F, Touyz RM. Adipocyte-specific mineralocorticoid receptor overexpression in mice is associated with metabolic syndrome and vascular dysfunction: Role of redox-sensitive pkg-1 and rho kinase. 2016; 65(8):2392-403.
6. Hood KY, Montezano AC, Harvey AP, Nilsen M, MacLean MR, Touyz RM. Nicotinamide adenine dinucleotide phosphate oxidase-mediated redox signaling and vascular remodeling by 16alpha-hydroxyestrone in human pulmonary artery cells: Implications in pulmonary arterial hypertension. *Hypertension*. 2016; 68(3):796-808.
7. Persson C, Kappert K, Engstrom U, Ostman A, Sjoblom T. An antibody-based method for monitoring in vivo oxidation of protein tyrosine phosphatases. *Methods*. 2005;35:37-43.
8. Bell D, Zhao Y, McCoy FP, Devine A, McDermott BJ. Expression of the counter-regulatory peptide intermedin is augmented in the presence of oxidative stress in hypertrophied cardiomyocytes. *Cell Physiol Biochem*. 2008;21:409-420.
9. MacLean MR, Deuchar GA, Hicks MN, Morecroft I, Shen S, Sheward J, Colston J, Loughlin L, Nilsen M, Dempsie Y, Harmar A. Overexpression of the 5-hydroxytryptamine transporter gene: Effect on pulmonary hemodynamics and hypoxia-induced pulmonary hypertension. *Circulation*. 2004;109:2150-2155.
10. Morecroft I, Pang L, Baranowska M, Nilsen M, Loughlin L, Dempsie Y, Millet C, MacLean MR. In vivo effects of a combined 5-ht1b receptor/sert antagonist in experimental pulmonary hypertension. *Cardiovasc Res*. 2010;85:593-603.
11. Keegan A, Morecroft I, Smillie D, Hicks MN, MacLean MR. Contribution of the 5-ht(1b) receptor to hypoxia-induced pulmonary hypertension: Converging evidence using 5-ht(1b)-receptor knockout mice and the 5-ht(1b/1d)-receptor antagonist gr127935. *Circ Res*. 2001;89:1231-1239.
12. Johansen AK, Dean A, Morecroft I, Hood K, Nilsen M, Loughlin L, Anagnostopoulou A, Touyz RM, White K, MacLean MR. The serotonin transporter promotes a pathological estrogen metabolic pathway in pulmonary hypertension via cytochrome p450 1b1. *Pulm Circ*. 2016;6:82-92.
