# Supplementary material for: Serotonin Signaling Through the 5-HT1B Receptor and NADPH Oxidase 1 in Pulmonary Arterial Hypertension
Source: Arterioscler Thromb Vasc Biol. 2017 Jun 21;37(7):1361–70. doi: 10.1161/ATVBAHA.116.308929 (PMC5478178; doi:10.1161/ATVBAHA.116.308929)
Supplement: Supplementary file 3 [file atv-37-1361-s003.pdf]

Supplemental Material

Serotonin Signaling Through the 5-HT_{1B} Receptor and NADPH Oxidase 1 in Pulmonary Arterial Hypertension.

Katie Y Hood PhD, Kirsty M Mair PhD, Adam P Harvey PhD, Augusto C Montezano PhD, Rhian M Touyz MD PhD*, Margaret R MacLean PhD*.
**Joint senior authors*

Institute of Cardiovascular and Medical Sciences, University of Glasgow, United Kingdom.

Table I. PAH Patient and non-PAH patient information.

Patient Group	Sex	Age (years)	Disease Status
Non-PAH	Female	57	COPD
		58	Mild emphysema
		59	Squamous cell carcinoma
		64	Lung carcinoma
		70	Lobectomy
PAH	Female	30	HPAH (R899X)
		33	IPAH
		39	IPAH
		41	HPAH (N903S)
		53	IPAH

Pulmonary artery smooth muscle cell subject origin and characteristics. Known characteristics of subjects from whom cells were derived. COPD, chronic obstructive pulmonary disease; HPAH, heritable pulmonary arterial hypertension (gene mutation in parenthesis); IPAH, idiopathic pulmonary arterial hypertension.

Table II. Human primers for real time PCR analysis.

**Human Gene**	**Forward Primer**	**Reverse Primer**
GAPDH	GAGTCAACGGATTTGGTCGT	TTGATTTTGGAGGGATCTCG
MMP2	TCTCCTGACATTGACCTTGGC	CAAGGTGCTGGCTGAGTAGATC
MMP9	TTGACAGCGACAAGAAGTGG	GCCATTCACGTCGTCCTTAT
Nox1	TCACCAATTCCCAGGATTGA	TGTGGTCTGCACACTGGAAT
GPx	AGTCGGTGTATGCCTTCTCG	TTGAGACAGCAGGGCTCGAT

Human primers targeted to the above genes were designed using Primer 3 software, and were used to assess gene expression in control hPASMCs and PAH-hPASMCs.

Supplemental Figure I

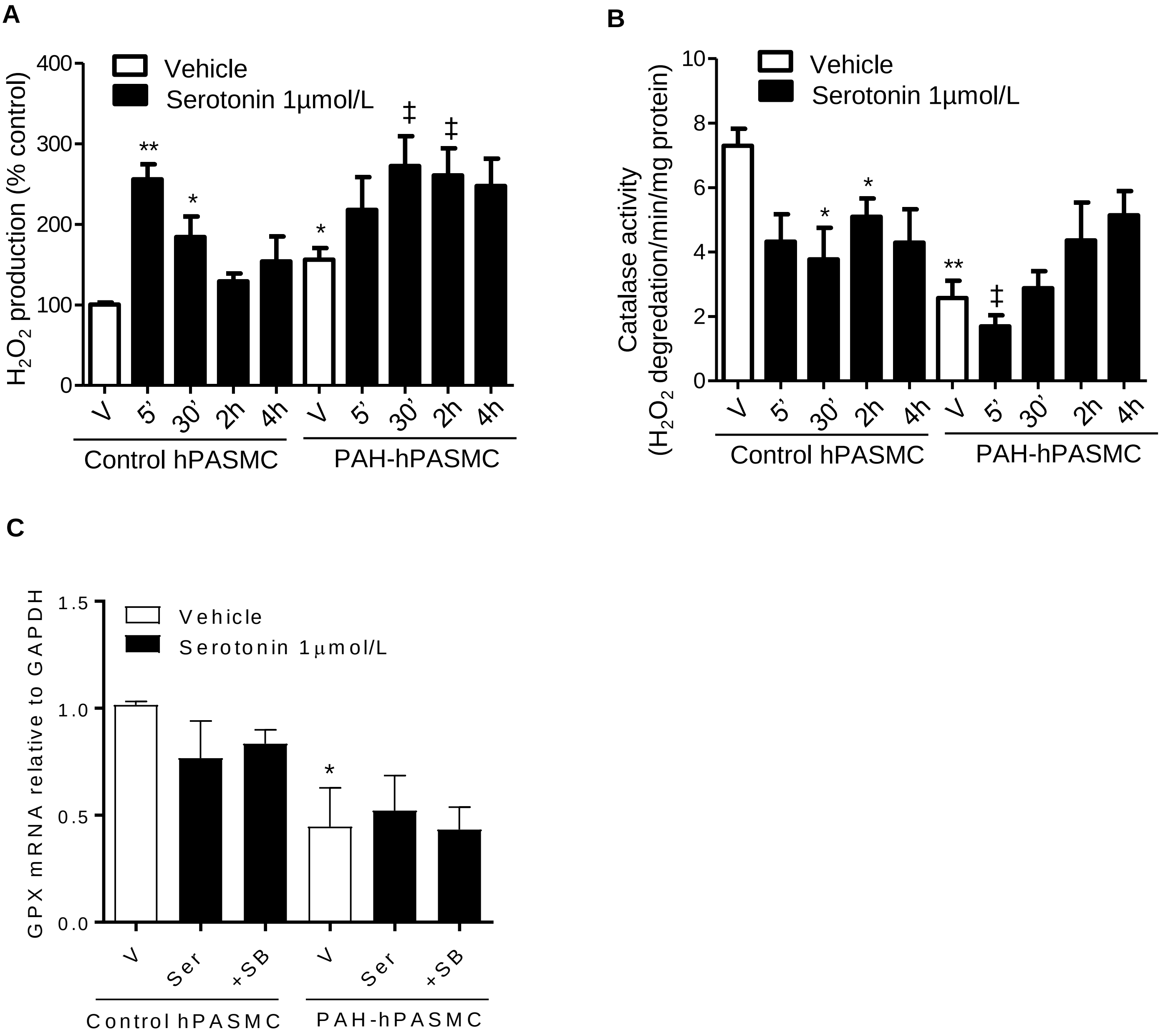

Supplemental Figure I: Effect of serotonin on H_2O_2 and antioxidants, catalase and glutathione. H_2O_2 production (A) and catalase activity (B) in hPASMCs was measured by Amplex Red assay in cells exposed to serotonin for 5 minutes to 4 hours. Data are expressed as RLU/µg protein corrected to standard curve and expressed as percentage of vehicle conditions. Glutathione mRNA expression (C) in hPASMCs. mRNA expression is relative to GAPDH. Results are mean ± SEM of 5-6 experiments, in triplicate. *$p<0.05$, **$p<0.01$ vs. vehicle control hPASMC; ‡$p<0.05$ vs. vehicle PAH-hPASMCs determined by ANOVA with Tukey's post-hoc test. V= vehicle; Ser= serotonin; SB= SB224289.

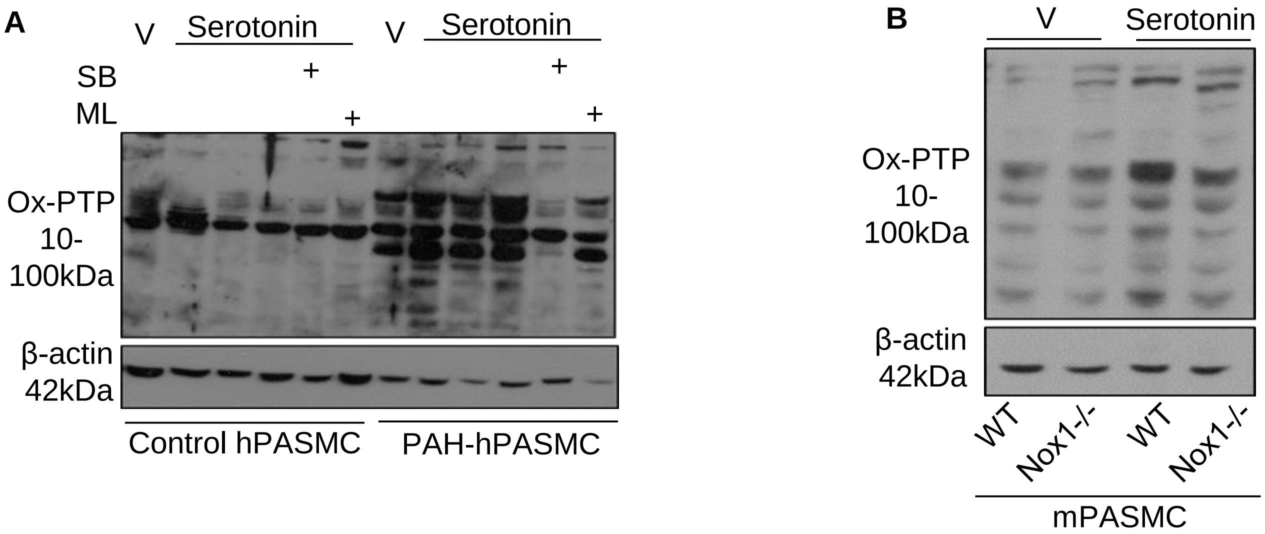

Supplemental Figure II: Serotonin promotes oxidation of PTPs. Irreversible oxidation of PTPs using the oxPTP antibody, in response to serotonin in the presence or absence of 5-HT_{1B}R antagonist SB224289 or Nox1 inhibitor, ML171 in hPASMCs (A) and in WT and Nox1-/- mPASMCs (B). Values are mean ± SEM of 4 experiments. Protein expression is relative to β-actin. Representative blots corresponding to Figure 3A and 3B. V= vehicle; SB= SB224289; ML= ML171; WT= wild-type.

Supplemental Figure III

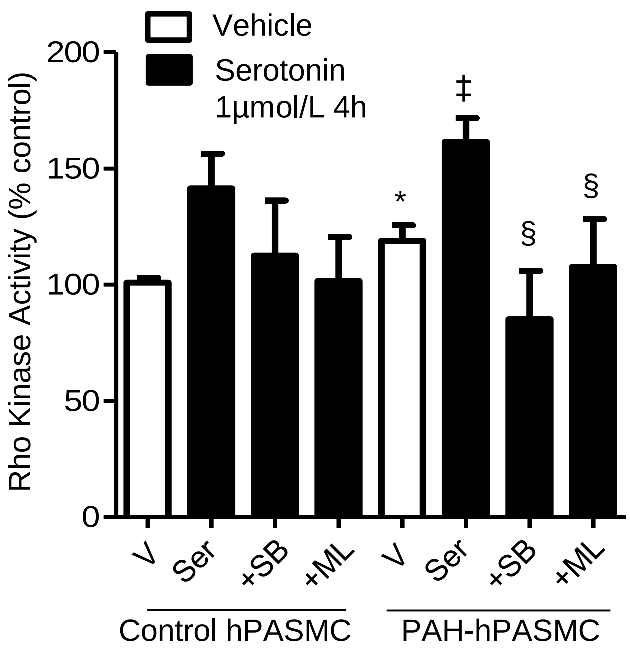

Supplemental Figure III: Role of 5-HT$_{1B}$R and Nox1 in serotonin-mediated vascular contraction. Rho Kinase activity assay was used to assess serotonin-induced Rho kinase activity in hPASMCs. Results are expressed as mean±SEM of 5 experiments relative to protein concentration. *p<0.05 vs vehicle control hPASMCs; ‡p<0.05 vs vehicle PAH-hPASMCs; §p<0.05 vs treated PAH-hPASMCs determined by ANOVA with Tukey's post-hoc test. V= vehicle; Ser= serotonin; SB= SB224289; ML= ML171.

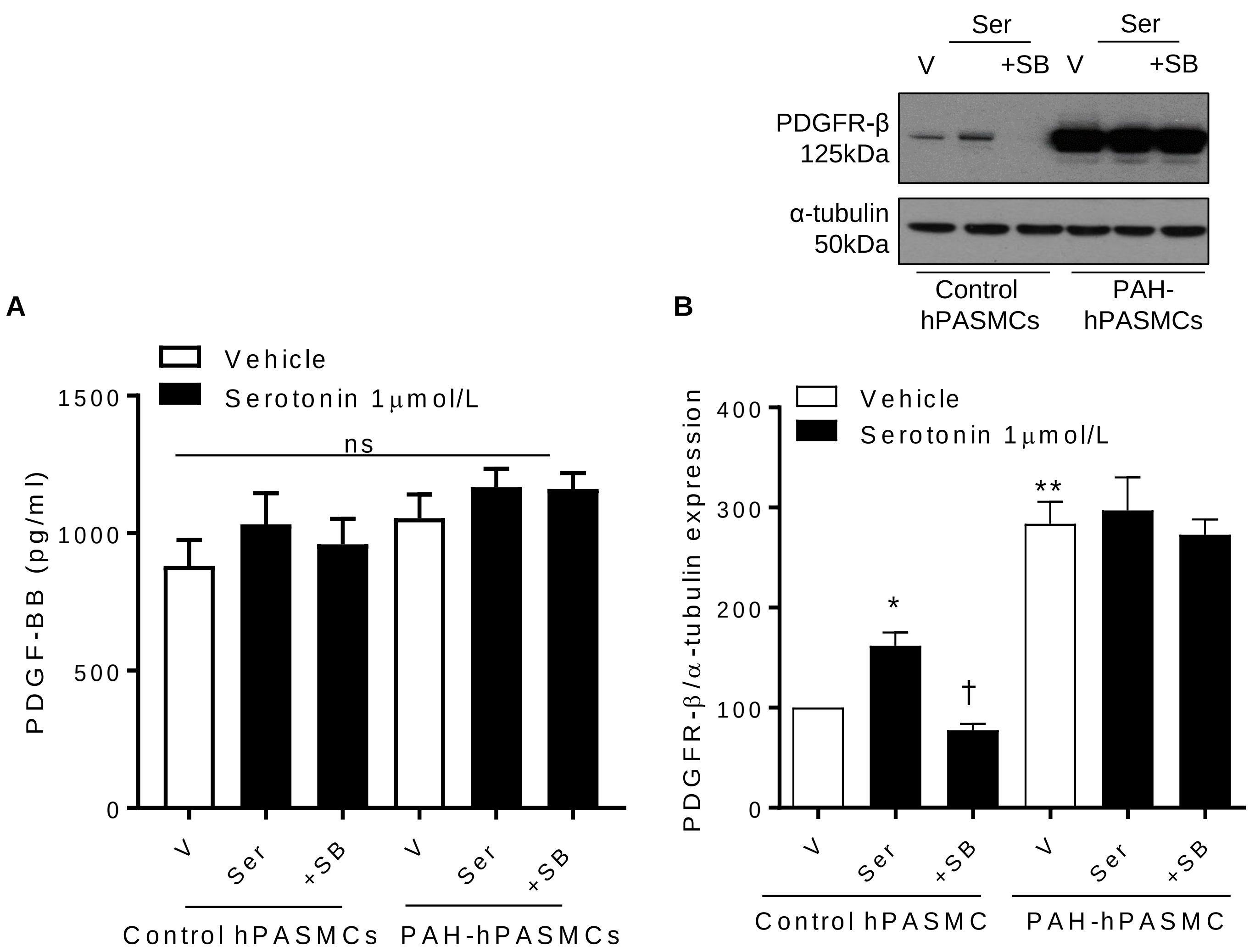

Supplemental Figure IV: Effect of serotonin on secretion of PDGF-BB and PDGFR-β expression. Secretion of PDGF-BB into hPASMC culture supernatant assessed by ELISA (A). Expression of PDGFRB in response to serotonin in hPASMCs (B). Results are mean ±SEM, n=6 per group. ns: not significant. *p<0.05; **p<0.01 vs vehicle control hPASMCs; †p<0.05 vs serotonin-treated control hPASMCs. V= vehicle; Ser= serotonin; SB= SB224289.

Supplemental Figure V

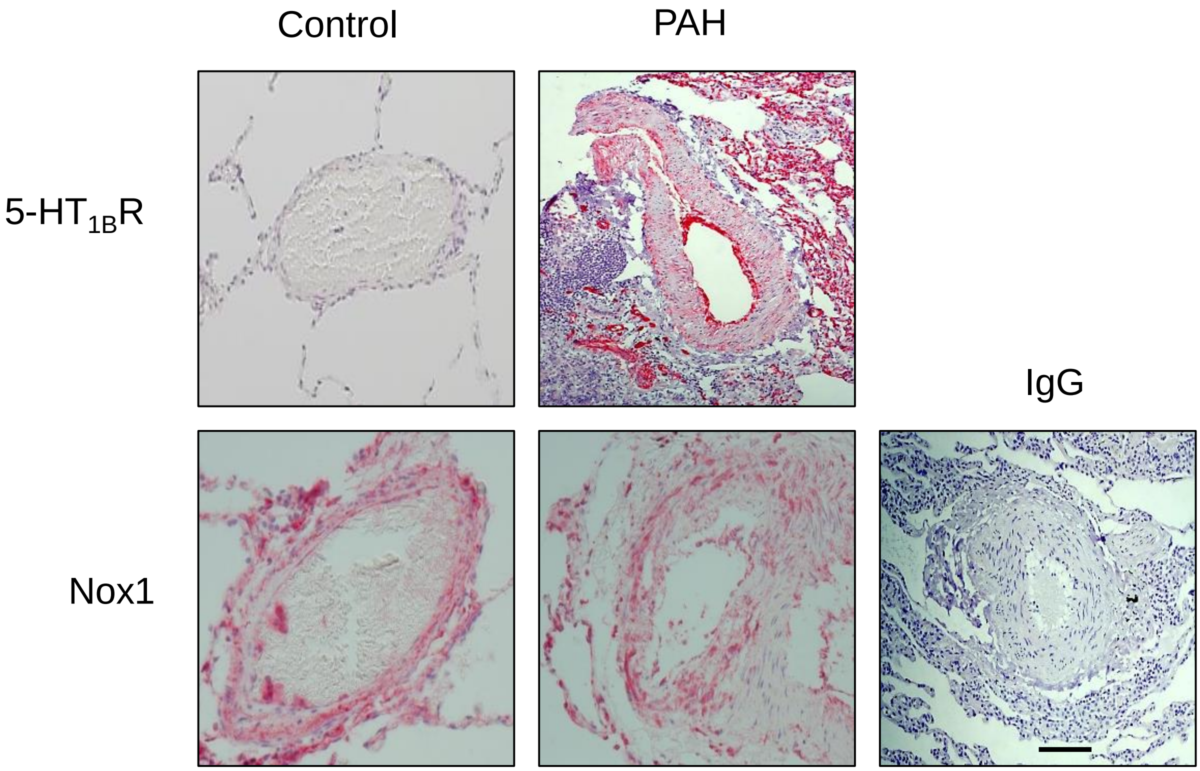

Supplemental Figure V: 5-HT$_{1B}$ receptor and Nox1 staining in human control and PAH lung sections. 5-HT$_{1B}$ receptor and Nox1 staining in human pulmonary arteries in lung sections from controls and PAH patients, counterstained with haematoxylin. Scale bars = 100 microns. n=2-5 per group.

Supplemental Figure VI

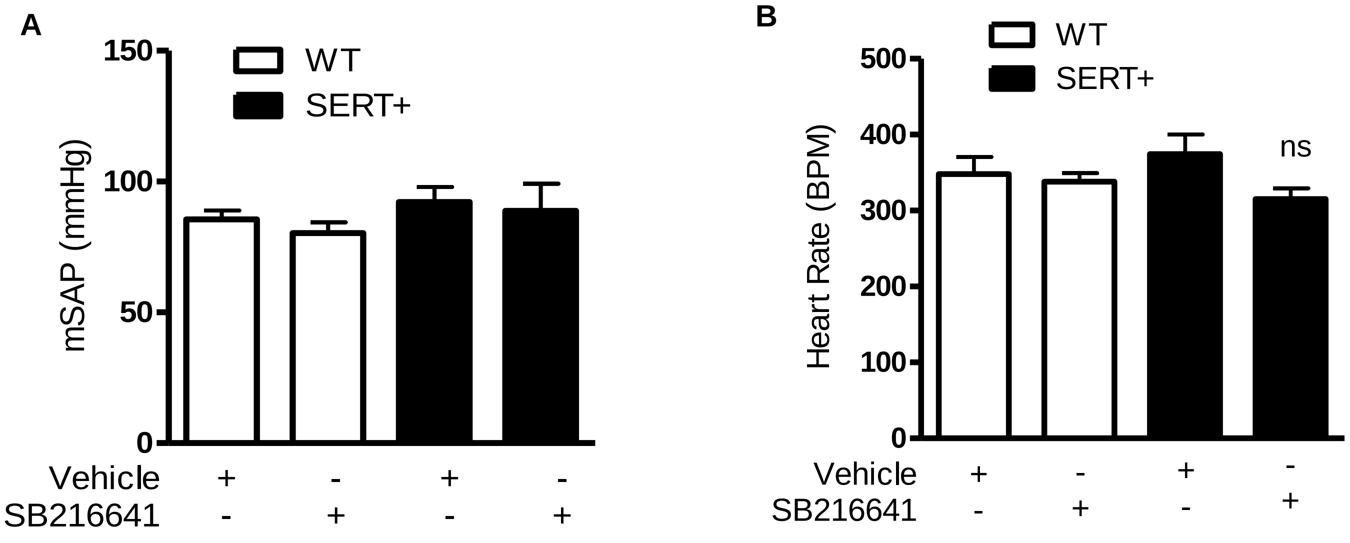

Supplemental Figure VI: Haemodynamic assessment of pulmonary hypertension in female WT and SERT+ mice treated with SB216641. Mean systemic arterial pressure (mSAP) (A) and heart rate (HR) (B). Results are mean ±SEM, n=8-10 per group. ns: not significant. WT = wild-type, BPM = beats per minute.

Supplemental Figure VII

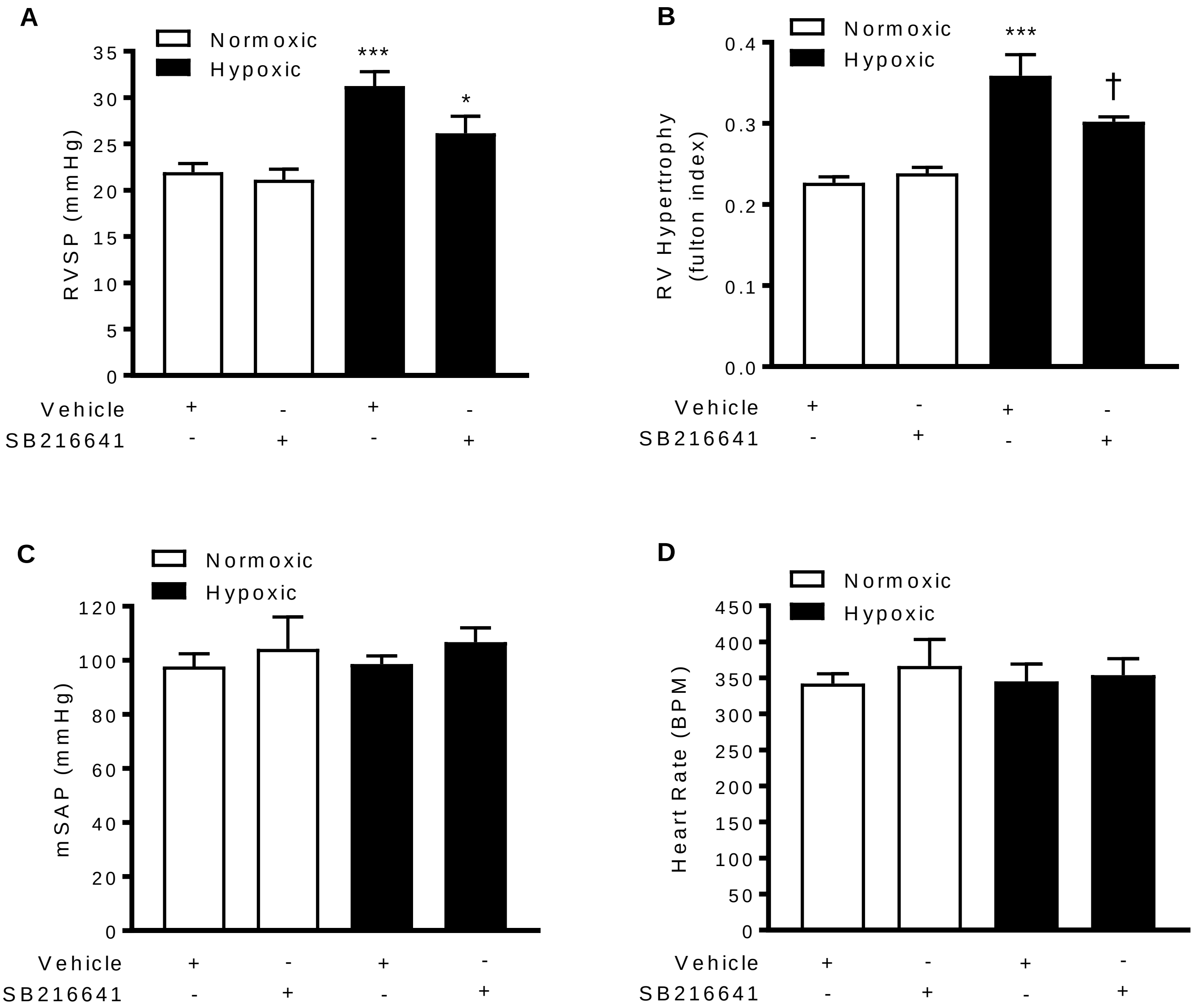

Supplemental Figure VII: Haemodynamic assessment of pulmonary hypertension in female normoxic and hypoxic mice treated with SB216641. Right ventricular systolic pressure (RVSP) (A), right ventricular hypertrophy (B), mean systemic arterial pressure (mSAP) (C) and heart rate (HR) (D). Results are mean ±SEM, n=8-10 per group. *p<0.05; ***p<0.001 versus normoxic vehicle treated mice; †p<0.05 versus hypoxic vehicle treated mice. BPM = beats per minute.

Supplemental Figure VIII

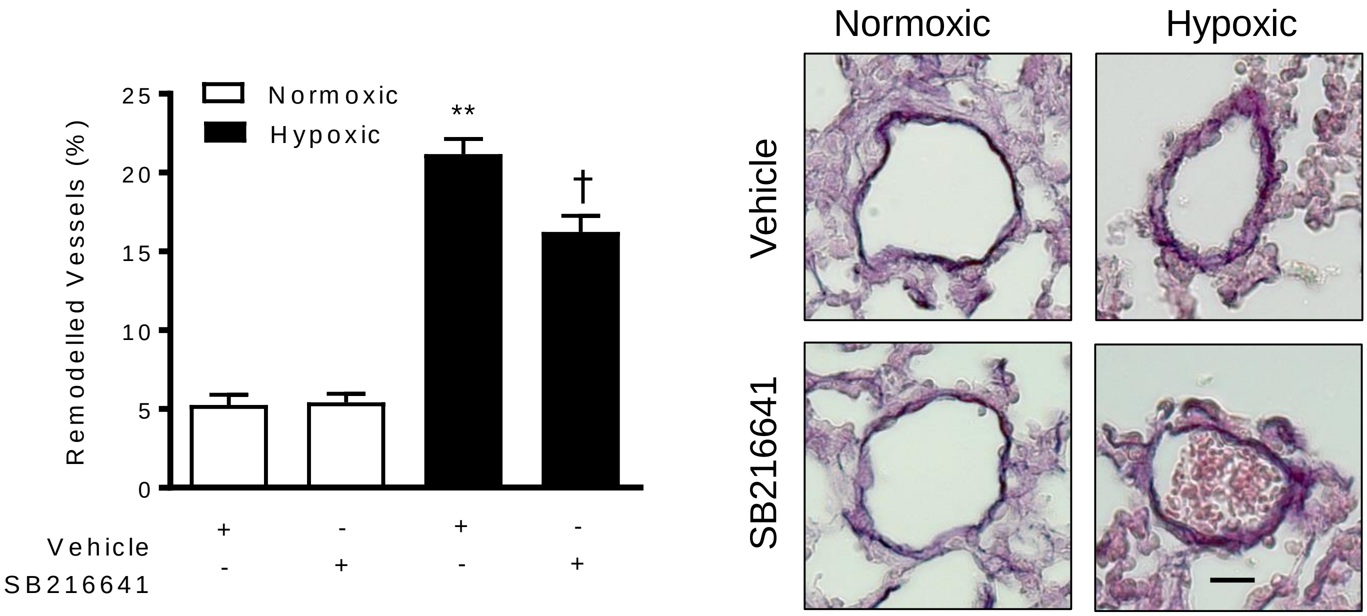

Supplemental Figure VIII: Pulmonary vascular remodelling in female normoxic and hypoxic mice treated with SB216641. Effects of SB216641 on percentage of pulmonary vascular remodeling in distal pulmonary arteries in female normoxic and hypoxic mice with representative images (right) of pulmonary arteries (Elastin Van Giesen stain; scale bars = 50 microns). Results are mean ±SEM, n=8 per group. **$p<0.01$ vs Normoxic vehicle, †$p<0.05$ versus hypoxic vehicle-treated mice, determined by 2-way ANOVA with Tukey's post-hoc test.

Supplemental Figure IX

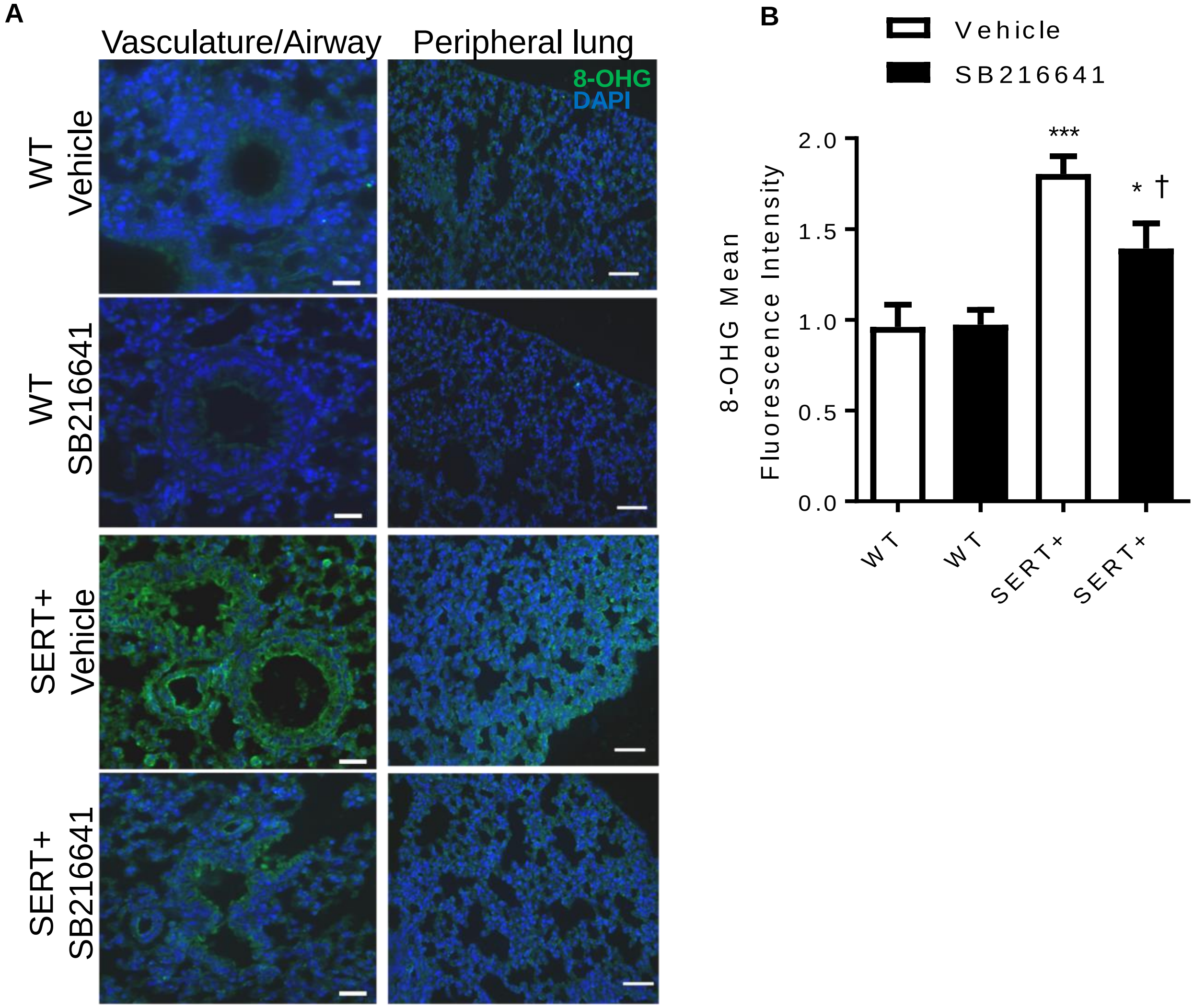

Supplemental Figure IX: Oxidative stress marker,8-hydroxyguanosine, is increased in the lungs of female SERT+ mice. Lung sections obtained from wild-type (WT) and SERT+ mice treated with the 5-$HT_{1B}R$ antagonist (SB216641) or vehicle, were immunostained for the oxidative-stress marker 8-hydroxyguanosine (8-OHG). Sections were counterstained with the nuclear stain DAPI (4′,6-diamidino-2-phenylindole). Scale bar: 50 µm (A). 8-OHG mean fluorescence intensity was measured in lung sections using ImageJ software; n = 4 (B). *p<0.05; ***p<0.01 vs WT vehicle; †p<0.05 vs SERT+ vehicle, determined by 2-way ANOVA with Tukey's post-hoc test. WT = wild-type, SERT+ = serotonin transporter overexpression. SB216641 = 5-$HT_{1B}R$ antagonist.

Supplemental Figure X

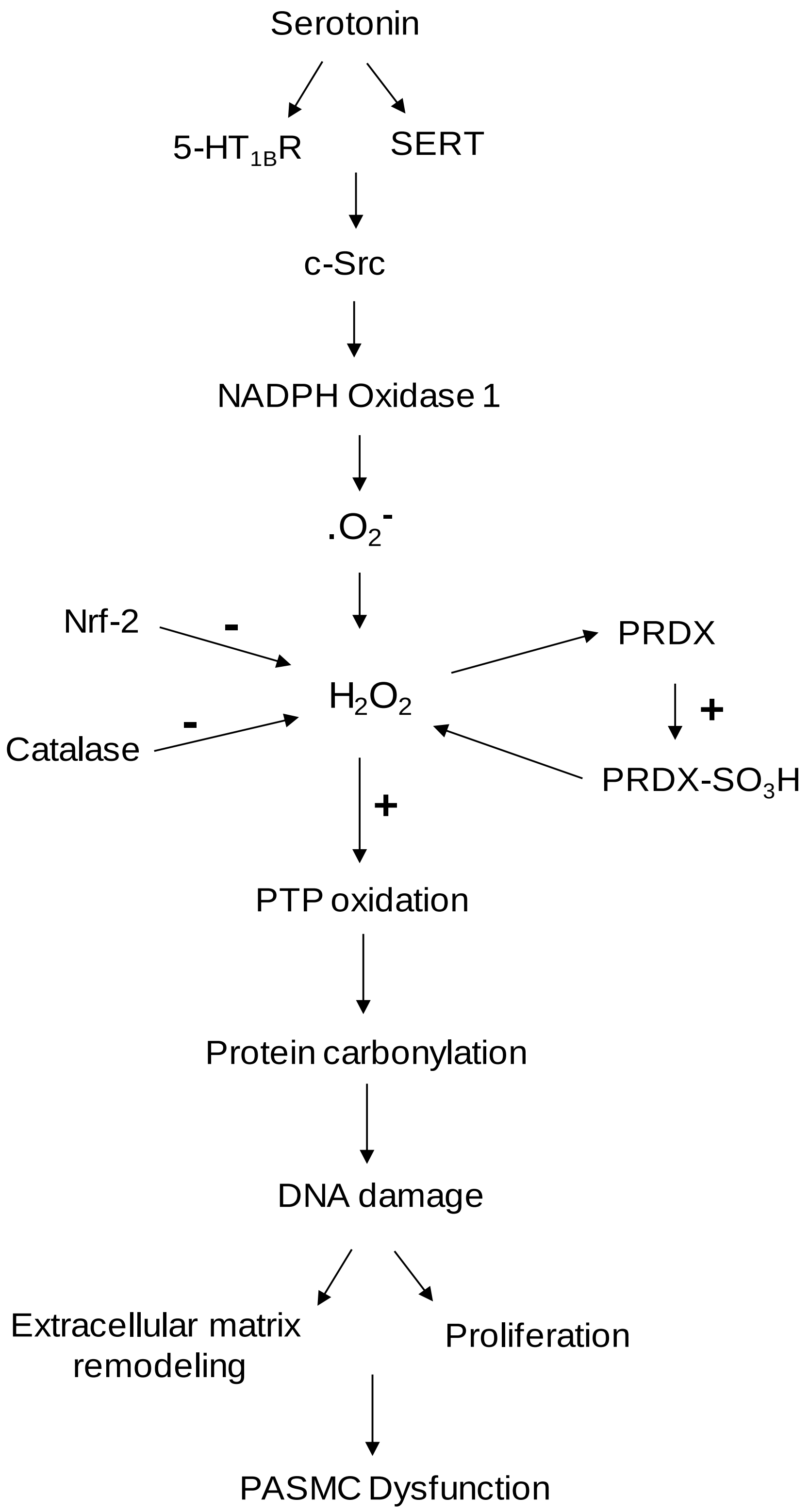

Supplemental Figure X: Schematic of putative role of serotonin in hPASMCs. Actions of serotonin in PASMCs are mediated not only via the 5-HT_{1B}R and SERT, but also involve the activation of Noxs, particularly Nox1, which leads to .O_2- and H_2O_2 production. Excessive ROS production coupled with impaired Nrf2-mediated antioxidant mechanisms in response to serotonin may promote oxidation of PTPs and total protein carbonylation and proteins involved in mitogenic and fibrotic effects, leading to deleterious oxidative stress and pulmonary vascular remodeling. + activation; - inhibition.
